# AI-assisted single-cell transcriptomics defines immunosuppressive ecosystem states and CD274-centered communication networks in myelodysplastic syndrome

**DOI:** 10.3389/fcell.2026.1897026

**Published:** 2026-10-30

**Authors:** Yacong Shao, Wenwei Zhu, Kai Wang, Changgui Li, Jiekai Li, Hailin Chen, Yongming Zhou

**Affiliations:** 1 Department of Hematology, Yueyang Hospital of Integrated Traditional Chinese and Western Medicine, Shanghai University of Traditional Chinese Medicine, Shanghai, China; 2 Department of Hematology, Shanghai Municipal Hospital of Traditional Chinese Medicine, Shanghai, China

**Keywords:** AI-assisted analysis, cancer ecosystem states, immune microenvironment, myelodysplastic syndrome, single-cell transcriptomics

## Abstract

**Background:**

Defining the transcriptional ecosystem states that govern immune dysfunction in myelodysplastic syndromes (MDS) is critical for identifying mechanistically grounded therapeutic targets in this heterogeneous hematopoietic malignancy. The MDS bone marrow immune ecosystem undergoes profound disease-associated remodeling that shifts from immune-mediated hematopoietic suppression in early disease to a globally immunosuppressive state that enables malignant clone expansion, yet the ecosystem-state architecture underlying this transition remains incompletely characterized.

**Methods:**

We deployed an AI-assisted single-cell transcriptomic analytical pipeline—integrating unsupervised Leiden machine learning clustering, multi-modal dimensionality reduction (UMAP, t-SNE, PCA), pathway module scoring, and ligand-receptor inference—on public bone marrow mononuclear cell datasets (GSE241417 as primary MDS scRNA-seq cohort; GSE120221 as external healthy bone marrow reference) to define immune ecosystem states across 11 transcriptionally distinct cell populations. Disease-associated T/NK-cell ecosystem state remodeling, immunosuppressive cytokine and checkpoint programs, and CD274-centered intercellular communication networks were systematically characterized. Key immune regulatory genes (IL10, TGFB1, CD274, FOXP3) were validated by RT-PCR in MDS-derived cell lines, and CD274 perturbation experiments were performed to investigate its regulatory role in shaping immunosuppressive ecosystem programs.

**Results:**

Eleven transcriptionally distinct immune ecosystem states were resolved within the MDS bone marrow microenvironment, with disease-associated redistribution toward cytotoxic and exhaustion-like T/NK programs. Pathway analysis revealed enrichment of T cell activation, interferon response, and cytokine signaling across ecosystem states. CD274-high cells were associated with elevated TGFB, checkpoint, and immunoregulatory module scores, while computationally inferred CD274-PDCD1 communication coexisted with TGFB1-TGFBR, LGALS9-HAVCR2, and IL10-related axes, suggesting a multi-layered immunosuppressive network. RT-PCR confirmed increased IL10, TGFB1, and CD274 expression in both MDS-derived cell lines; FOXP3 was significantly increased in SKM-1 but not significantly changed in MUTZ-1. CD274 perturbation was associated with marked IL-10 changes in SKM-1, whereas knockdown produced little apparent IL-10 reduction in MUTZ-1; FOXP3 responses were also cell-line dependent.

**Conclusion:**

This study defines the MDS bone marrow immune ecosystem at single-cell resolution using AI-assisted transcriptomic analysis, identifying CD274 as a molecule associated with immunosuppressive ecosystem programs. The findings support a hypothesis-generating model in which CD274-associated signaling may coexist with cytokine and checkpoint communication networks in MDS and may inform future ecosystem-focused therapeutic studies. Further validation using primary patient-derived immune subsets and protein-level assays is required.

## Introduction

Cancer ecosystems are defined by the complex interplay between malignant cells, non-malignant stromal and immune populations, and the microenvironmental signals that govern their collective functional behavior (Hanahan, 2022). In hematopoietic malignancies such as myelodysplastic syndromes (MDS), characterizing the transcriptional states of bone marrow immune ecosystem components is increasingly recognized as essential for understanding disease progression and identifying therapeutically actionable vulnerabilities (Kouroukli et al., 2022). MDS represents a heterogeneous group of clonal hematopoietic stem cell disorders characterized by ineffective hematopoiesis, peripheral blood cytopenias, dysplastic bone marrow morphology, and variable risk of transformation to acute myeloid leukemia (AML) (Kouroukli et al., 2022). The annual incidence of MDS is approximately 4–5 cases per 100,000 individuals, with prevalence increasing substantially with age, predominantly affecting individuals over 60 years old (Neukirchen et al., 2011). Despite advances in genomic and molecular landscape profiling through next-generation sequencing technologies, the ecosystem-level mechanisms driving disease initiation, progression, and transformation remain incompletely understood.

The MDS bone marrow immune ecosystem undergoes profound disease-associated remodeling that reflects the dynamic interplay between malignant clones and the surrounding immune compartment (Montes et al., 2019; Chen et al., 2018). In early-stage or lower-risk MDS, the ecosystem is characterized by aberrant T cell activation and cytotoxic programs that suppress normal hematopoiesis, sharing certain immunopathological features with aplastic anemia (Glenthøj et al., 2016). As disease progresses, the ecosystem shifts toward a globally immunosuppressive state in which malignant clones co-opt immune regulatory networks to promote immune escape, clonal expansion, and progression risk (Shallis et al., 2018). This ecosystem-level biphasic shift highlights the need for transcriptional ecosystem state characterization that extends beyond tumor-cell-centric classifications to capture the microenvironmental composition and communication patterns that shape disease behavior and therapeutic vulnerability.

Multiple components of both innate and adaptive immunity are aberrantly activated within the MDS bone marrow ecosystem (Liu et al., 2023). T cell dysfunction is a hallmark ecosystem feature, encompassing altered subset distribution, functional impairment, and exhaustion-like phenotypes (R et al., 2024). Within the MDS ecosystem, CD4^+^ T cell depletion, regulatory T cell (Treg) expansion, and upregulation of inhibitory immune checkpoint molecules—including programmed cell death protein-1 (PD-1), cytotoxic T-lymphocyte-associated protein 4 (CTLA-4), T-cell immunoglobulin and mucin-domain containing-3 (TIM-3), and lymphocyte-activation gene 3 (LAG3)—have been documented (Yang et al., 2014; Boddu et al., 2018). The PD-1/PD-L1 (CD274) axis shapes ecosystem-level immunosuppression by suppressing T cell receptor-mediated proliferation and cytokine release, thereby enabling malignant clone survival within the immune ecosystem (Yang et al., 2022).

Beyond T cells, additional immune ecosystem components contribute to the complex immunological landscape of MDS. B cell abnormalities including altered differentiation patterns and antibody production defects, natural killer (NK) cell dysfunction characterized by impaired cytotoxic capacity, and pathological expansion of myeloid-derived suppressor cells (MDSCs) collectively generate an immunosuppressive ecosystem niche (Li and Calvi, 2017; Kordasti et al., 2016; Cheng et al., 2019). The secretion of immunosuppressive factors such as S100A9, interleukin-10 (IL-10), and transforming growth factor-beta (TGF-β) by diverse ecosystem components creates a coordinated, multi-cellular immunosuppressive program that both damages normal hematopoiesis and protects malignant clones from immune clearance.

The advent of single-cell RNA sequencing (scRNA-seq) combined with computational and unsupervised machine-learning approaches has enabled high-resolution characterization of cellular transcriptional states (Liu et al., 2022; Luecken and Theis, 2019). In MDS research, these computational approaches allow comprehensive characterization of individual cell-type transcriptional states, identification of rare ecosystem subpopulations, reconstruction of differentiation trajectories, and mapping of cell-cell communication networks that coordinate ecosystem behavior. Recent scRNA-seq studies have revealed distinct hematopoietic stem cell hierarchies in MDS, uncovered aberrant differentiation programs, and identified molecular signatures associated with disease risk stratification and treatment response (Ganan-Gomez et al., 2022; Oetjen et al., 2018). However, systematic computational characterization of the MDS immune microenvironment—particularly integrating lymphoid state remodeling, immunosuppressive checkpoint programs, and CD274-centered ligand-receptor communication networks—remains incompletely defined.

Several critical questions in MDS immune ecosystem biology remain unanswered. First, the transcriptional state composition of the MDS bone marrow immune ecosystem and its disease-associated remodeling patterns require systematic computational definition. Second, the mechanisms underlying immune checkpoint upregulation and their consequences across different ecosystem cell populations require characterization. Third, the cell-cell communication networks that coordinate immunosuppression and shape ecosystem state transitions need reconstruction and functional context. Fourth, because malignant hematopoietic and non-malignant immune cells may share overlapping immune regulatory transcripts in scRNA-seq data, careful ecosystem-level interpretation is required when assigning disease-associated gene expression to specific cellular sources. Addressing these knowledge gaps is essential for developing ecosystem-informed immunotherapeutic strategies to improve outcomes for MDS patients.

In this study, we performed single-cell transcriptomic analysis incorporating unsupervised Leiden clustering of bone marrow mononuclear cells from public MDS datasets to define the immune ecosystem state composition at single-cell resolution. Through integrated machine learning clustering, dimensionality reduction, pathway scoring, and ligand-receptor inference, we identified eleven distinct immune ecosystem states, characterized their transcriptional signatures, evaluated immune checkpoint expression patterns across ecosystem states, performed pathway enrichment analysis, and reconstructed the CD274-centered communication network shaping MDS immunosuppressive ecosystem programs. Key immune regulatory genes were validated through quantitative RT-PCR in MDS-derived cell lines, and CD274 perturbation experiments were used to explore its association with immunoregulatory programs in MDS-derived cell-line models.

## Methods

### Data acquisition and sample information for ecosystem state analysis

Single-cell RNA sequencing data were obtained from the Gene Expression Omnibus (GEO) database to enable ecosystem state characterization of MDS bone marrow. GSE241417 was used as the primary MDS scRNA-seq cohort for immune ecosystem state definition, and GSE120221 was used as an external healthy bone marrow reference dataset for descriptive disease-control ecosystem integration. Available clinical and sample-level metadata were extracted from GEO family records and summarized in Supplementary Table S1. The raw sequencing data were downloaded and processed to capture heterogeneous immune ecosystem cell populations within the MDS bone marrow microenvironment.

This study comprises two distinct components: (1) computational reanalysis of public scRNA-seq data from GEO datasets for ecosystem state definition, and (2) original *in vitro* functional experiments conducted in our laboratory to validate key ecosystem regulatory mechanisms. For the public GEO datasets, available metadata included sample identifiers, GEO accession numbers, disease status, sample source, and treatment or sampling context. Because detailed MDS subtype, IPSS-R risk category, age, sex, longitudinal treatment response, and clinical outcome information were incomplete in the public metadata, the ecosystem state analysis was conducted at the disease-context and sample-context levels rather than stratified by subtype, treatment stage, or outcome.

### Quality control and data processing

Raw sequencing data underwent rigorous quality control assessment to ensure data reliability for ecosystem state definition. Cells were filtered based on the following criteria: gene detection range of 3,000–7,000 genes per cell, UMI counts between 10,000 and 80,000 per cell, and mitochondrial gene content below 10%. These predefined filters were used to retain cells within the selected gene-count, UMI-count, and mitochondrial-content ranges. Because the relatively restrictive thresholds may influence cell retention and low-abundance populations, this is considered when interpreting the results. Batch effect analysis was performed to evaluate technical variation across the six independent sequencing batches, and sample correlation matrices were generated using Pearson correlation coefficients to assess within-group and between-group similarities across ecosystem state samples.

### AI-driven ecosystem state definition: dimensional reduction and clustering

Multiple complementary dimensionality-reduction techniques were employed to identify transcriptionally distinct immune ecosystem states. Principal Component Analysis (PCA) was performed to capture major sources of transcriptional variation, with the first 30 principal components retained for subsequent ecosystem state analysis. Normalization was performed using scran-based normalization, and the top 3,000 highly variable genes (HVGs) were selected for downstream analysis; mitochondrial and ribosomal gene content were regressed out as covariates. Batch correction was applied using Harmony on the PCA embedding prior to UMAP construction and Leiden clustering, before UMAP construction and Leiden clustering. Harmony was used to reduce dataset-associated variation; however, residual dataset-of-origin effects cannot be excluded.

T-distributed Stochastic Neighbor Embedding (t-SNE) and Uniform Manifold Approximation and Projection (UMAP) were applied independently as complementary two-dimensional visualization methods for the same underlying Leiden clustering results. Three-dimensional UMAP was additionally generated to provide enhanced spatial resolution of ecosystem state relationships. Unsupervised clustering using the Leiden machine learning algorithm identified 11 distinct immune ecosystem state clusters based on transcriptional similarity. Cluster assignments were validated through examination of canonical immune cell markers and biological interpretation in the context of MDS ecosystem biology.

### Ecosystem state cell type annotation and marker gene analysis

Ecosystem state cell type annotation was performed through systematic profiling of established immune cell markers. T cell ecosystem states were identified by CD3D expression, B cells by MS4A1 (CD20) together with additional B-lineage and antigen-presentation-associated markers (CD79A, CD79B, CD74, HLA-DRA), monocytes/macrophages by CD14, helper T cells by CD4, and NK cells by NKG7. Cell-type annotation was based on combined marker patterns rather than a single marker alone. In this study, the term ‘ecosystem state’ is used operationally to describe transcriptionally distinct Leiden-defined populations/states within the broader bone marrow immune ecosystem and does not imply that each cluster is an independently validated biological ecosystem. Comprehensive marker gene expression matrices were generated for all 11 ecosystem state clusters, with expression levels visualized through dot plots, violin plots, and heatmaps. Hierarchical clustering analysis was performed to reveal transcriptional relationships between ecosystem state cell types and identify potential developmental trajectories within the immune ecosystem.

### Differential gene expression and pathway enrichment across ecosystem states

Differential gene expression analysis was conducted to identify ecosystem state-specific transcriptional signatures. For each cluster comparison, genes with log2 fold change >2 and adjusted p-value <0.05 (Bonferroni correction) were considered significantly differentially expressed. Volcano plots and MA plots were generated to visualize expression changes and identify highly significant ecosystem state marker genes. In this study, MDS-associated genes were operationally defined as genes showing differential expression between MDS-derived or MDS-associated cell states and control/reference cells, or genes enriched in MDS-associated ecosystem state clusters.

Pathway enrichment analysis was performed using established gene set databases to identify biological processes and signaling pathways dysregulated across MDS ecosystem states. Enrichment scores were calculated for immune-related pathways including T cell activation, B cell differentiation, innate immune response, inflammatory signaling, and cytokine production. Gene-pathway interaction networks were constructed to illustrate functional connections between immune ecosystem cell types and identify key regulatory nodes within the MDS immune ecosystem.

### Intercellular communication network reconstruction

Cell-cell communication networks within the MDS immune ecosystem were inferred through analysis of ligand-receptor pair expression across identified ecosystem state cell types. Communication scores were estimated from the mean transcript expression of selected cognate ligand-receptor pairs in annotated sender and receiver populations. This expression-based analysis was used as an exploratory inference rather than as direct evidence of functional signaling. Chord diagrams and network graphs were generated to visualize inferred interaction strengths and directional communication patterns between major immune ecosystem populations. These analyses were based on transcriptome-derived ligand and receptor expression; therefore, the resulting axes are described throughout as computationally inferred communication patterns and require experimental validation.

### Disease-control ecosystem integration and T/NK-cell state analysis

To assess disease-associated immune ecosystem remodeling, we integrated the MDS scRNA-seq dataset GSE241417 with the external healthy bone marrow reference dataset GSE120221. After quality control, normalization, highly variable gene selection, dimensionality reduction, and batch correction, MDS and healthy bone marrow cells were embedded into a shared UMAP space representing the combined ecosystem state landscape. Major immune cell types were annotated using canonical lineage markers, and sample-level cell-type proportions were compared descriptively using stacked bar plots, dumbbell plots, and PCA based on immune-cell composition. Cell-type-resolved gene-expression patterns and immune module scores—including inflammatory response, interferon response, T-cell activation, exhaustion-like state, and antigen-presentation programs—were further calculated to evaluate disease-associated ecosystem state changes.

To explore lymphoid ecosystem dysfunction, T/NK cells were extracted and reanalyzed separately. T/NK ecosystem subpopulations were annotated as CD4-like T cells, CD8 cytotoxic T cells, exhaustion-like T cells, Treg-like cells, NK/NKT-like cells, and intermediate T/NK states based on marker genes and functional signatures. Cytotoxicity, T-cell activation, exhaustion-like, and Treg-like module scores were used to characterize the functional-state distribution of T/NK ecosystem cells. Because healthy controls were derived from an external dataset, MDS-control comparisons were performed as descriptive ecosystem analyses.

### Immunosuppressive program mapping and CD274-Centered communication analysis

To localize key immunoregulatory molecules at single-cell ecosystem resolution, IL10, TGFB1, CD274, and FOXP3 expression was visualized on the integrated UMAP and compared across major immune ecosystem cell types. Additional immune checkpoint, inflammatory, and antigen-presentation genes—including PDCD1, CTLA4, HAVCR2, LAG3, TIGIT, PDCD1LG2, CD80, CD86, S100A8, S100A9, CXCL8, TNF, IL6, and HLA-DRA—were summarized using dot plots and heatmaps. Cells were stratified into CD274-high and CD274-low groups according to the expression-based stratification used in the analysis, and immune regulatory module scores were compared between these groups. These comparisons were interpreted as associations with CD274 expression status rather than evidence of direct CD274-mediated regulation. Cell-type-resolved Spearman correlation analysis was performed to assess associations between CD274 and IL10, TGFB1, or FOXP3 expression across ecosystem states.

A focused ligand-receptor analysis was performed to evaluate CD274-centered immunosuppressive communication across the MDS ecosystem. Communication strength was estimated from average ligand and receptor transcript expression in predefined sender and receiver cell populations, with ligand-expressing populations treated as potential senders and receptor-expressing populations as potential receivers. Selected checkpoint and cytokine-related pairs—including CD274–PDCD1, PDCD1LG2–PDCD1, CD80/CD86–CTLA4, CD80/CD86–CD28, LGALS9–HAVCR2, IL10–IL10RA/IL10 R B, TGFB1–TGFBR1/TGFBR2, and IFNG–IFNGR1/IFNGR2—were analyzed. CD274-centered communication maps, Sankey/alluvial plots, and sender-receiver matrices were generated to visualize the distribution of CD274-related ecosystem signaling across sender and receiver cell populations.

### RT-PCR validation of immune regulatory genes in MDS cell lines

#### Cell culture

MDS-derived cell lines SKM-1 and MUTZ-1 were cultured in RPMI-1640 medium supplemented with 10% fetal bovine serum, 100 U/mL penicillin, and 100 μg/mL streptomycin at 37 °C in a humidified atmosphere with 5% CO_2_. Normal human bone marrow-derived CD34^+^ hematopoietic stem/progenitor cells (Primary Bone Marrow CD34^+^ Cells, Normal, Human; ATCC® PCS-800–012™) were commercially obtained from the American Type Culture Collection (ATCC, Manassas, VA, USA) and used as reference controls.

#### RNA extraction and cDNA synthesis

Total RNA was extracted from cell lines using TRIzol reagent (Invitrogen) according to the manufacturer’s protocol. RNA concentration and purity were assessed by NanoDrop spectrophotometry (A260/A280 ratio >1.8). One microgram of total RNA was reverse transcribed to cDNA using a PrimeScript RT Reagent Kit (Takara) with oligo (dT) primers in a 20 μL reaction volume.

Quantitative Real-Time PCR. Quantitative RT-PCR was performed on a CFX96 Real-Time PCR Detection System (Bio-Rad) using SYBR Green Master Mix (Applied Biosystems). The thermal cycling conditions were: initial denaturation at 95 °C for 10 min, followed by 40 cycles of 95 °C for 15 s and 60 °C for 60 s. Melting curve analysis was performed to verify primer specificity.

Primer Design and Sequences. Gene-specific primers were designed using Primer-BLAST software. For IL10 amplification, the forward primer was 5′-GAC​TTT​AAG​GGT​TAC​CTG​GGT​TG-3′ and reverse primer was 5′-TCA​CAT​GCG​CCT​TGA​TGT​CTG-3’ (132 bp). TGFB1 was amplified using forward primer 5′-GGC​CAG​ATC​CTG​TCC​AAG​C-3′ and reverse primer 5′-GTG​GGT​TTC​CAC​CAT​TAG​CAC-3’ (187 bp). CD274 primers were 5′-TGG​CAT​TTG​CTG​AAC​GCA​TTT-3’ (forward) and 5′-TGC​AGC​CAG​GTC​TAA​TTG​TTT​T-3’ (reverse) (156 bp). FOXP3 was quantified using forward primer 5′-GTG​GCC​CGG​ATG​TGA​GAA​G-3′ and reverse primer 5′-GGA​GCC​CTT​GTC​GGA​TGA​TG-3’ (143 bp). GAPDH served as internal reference (226 bp).

#### Plasmid construction and transfection

CD274 overexpression plasmid (CD274-OE) was constructed by cloning the full-length human CD274 cDNA into pcDNA3.1 (+) vector. CD274-specific siRNA (si-CD274) and scrambled control siRNA were synthesized commercially. SKM-1 and MUTZ-1 cells were transfected using Lipofectamine 3,000 (Invitrogen). Briefly, 2 × 10^5^ cells were seeded in 6-well plates and transfected with 2.5 μg plasmid DNA or 50 nM siRNA. Cells were harvested 48 h post-transfection for subsequent analysis.

#### Immunofluorescence staining and image quantification

For IL-10 and FOXP3 detection, cells were fixed with 4% paraformaldehyde, permeabilized with 0.1% Triton X-100, and blocked with 5% bovine serum albumin (BSA). Cells were incubated with primary antibodies: rabbit anti-IL-10 (1:200, Abcam), mouse anti-β-tubulin (1:500, Cell Signaling Technology), and rabbit anti-FOXP3 (1:200, Cell Signaling Technology). Fluorescence-conjugated secondary antibodies (Alexa Fluor 488 goat anti-rabbit IgG and Alexa Fluor 594 goat anti-mouse IgG, 1:500, Invitrogen) were applied, and nuclei were counterstained with DAPI. Images were acquired using a confocal laser scanning microscope (Zeiss LSM 880). For IL-10, background-corrected fluorescence intensity was measured and normalized to the control group. For FOXP3, the percentage of FOXP3-positive cells was calculated by counting at least 500 cells per condition across three independent experiments.

Statistical Analysis. All statistical analyses were performed using Python (version 3.8) with Scanpy, Seaborn, and SciPy packages. Bonferroni correction was used for differential gene expression analysis; Benjamini–Hochberg FDR correction was applied for pathway enrichment analysis; and permutation-based p-value correction was applied for ligand-receptor interaction scoring. For RT-PCR validation, normality was assessed by Shapiro-Wilk test, and group comparisons were performed using one-way ANOVA with Tukey’s *post hoc* test. Data are presented as mean ± SEM. Cell-level comparisons were treated as exploratory and were not interpreted as equivalent to independent patient-level replication; future larger cohorts should confirm key disease-control findings using sample-level pseudobulk or mixed-effects approaches. All analysis scripts have been deposited in a publicly accessible repository (see Data Availability) to ensure full computational reproducibility.

## Results

### Quality control and ecosystem state profiling pipeline

Available clinical and sample-level metadata from GEO records are summarized in Supplementary Table S1. GSE241417 was used as the primary MDS scRNA-seq cohort for immune ecosystem state definition, whereas GSE120221 was used only as an external healthy bone marrow reference dataset for descriptive disease-control ecosystem integration. Quality control analysis revealed robust sequencing metrics across all MDS samples (Figure 1A), with consistent gene detection (3,000–7,000 genes per cell) and UMI counts (10,000–80,000 per cell), while mitochondrial content remained low (<10%), indicating high-quality cellular data suitable for ecosystem state profiling. The gene-UMI relationship (Figure 1B) demonstrated typical single-cell sequencing characteristics with clear linear correlation, confirming technical reliability. Batch effect analysis (Figure 1D) revealed minimal technical variation across six batches, with comparable UMI count distributions. The sample correlation matrix (Figure 1E) indicated strong within-group correlations, particularly among MDS samples, while showing expected divergence between disease and control groups. UMAP dimensional reduction (Figure 1F) successfully separated cells into distinct ecosystem state clusters, with samples distributed across multiple cellular populations, suggesting heterogeneous immune ecosystem composition in the MDS bone marrow microenvironment.

**FIGURE 1:**
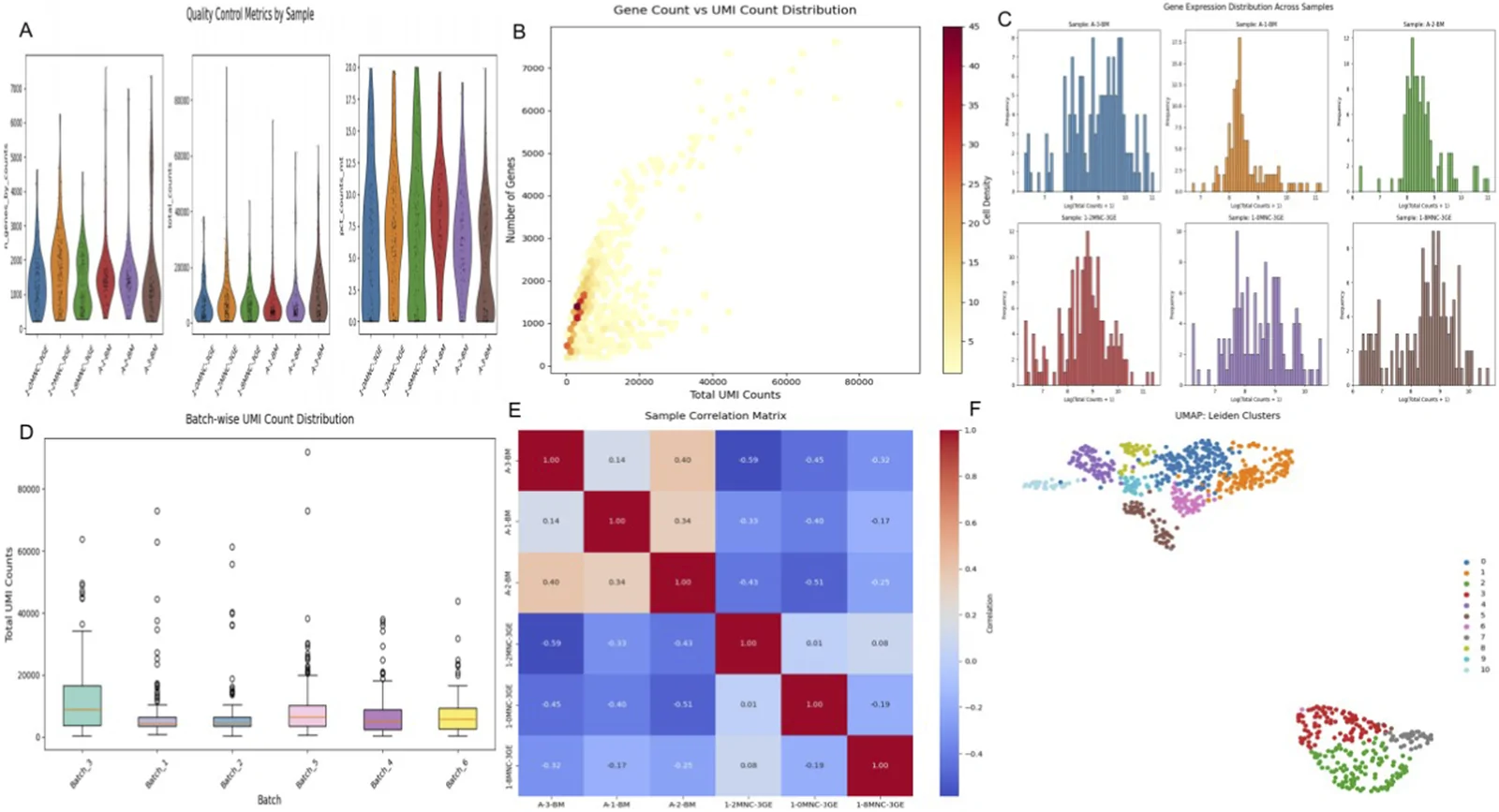
Quality control and initial ecosystem profiling. **(A)** Genes, UMI counts, and mitochondrial percentage across samples. **(B)** Gene–UMI relationship. **(C)** Sample-specific gene-expression distributions **(D)** Batch-wise UMI distributions. **(E)** Sample correlation matrix. **(F)** UMAP of Leiden clusters and sample distribution.

### AI-driven dimensionality reduction resolves eleven distinct immune ecosystem states

Multiple dimensionality-reduction approaches consistently identified eleven distinct immune ecosystem states within the MDS bone marrow. t-SNE visualization (Figure 2A) revealed 11 well-separated Leiden ecosystem state clusters with clear boundaries, indicating distinct transcriptional states. PCA biplot (Figure 2B) demonstrated that the top variable genes effectively captured biological variation across ecosystem states, with clusters separating along the first two principal components. UMAP analysis by sample origin (Figure 2C) showed that cells from different samples distributed across multiple ecosystem state clusters, suggesting shared cellular identities despite sample heterogeneity. The 3D UMAP visualization (Figure 2D) provided additional resolution of ecosystem state relationships in three-dimensional transcriptional space. PCA variance analysis (Figure 2E) indicated that the first two principal components captured approximately 7% of total variance; downstream clustering was performed on the top 30 PCs collectively, which together capture sufficient variance for robust neighbor-graph construction and ecosystem state definition. Key immune marker expression patterns (Figure 2F) across three timepoints confirmed distinct immune ecosystem state compositions.

**FIGURE 2:**
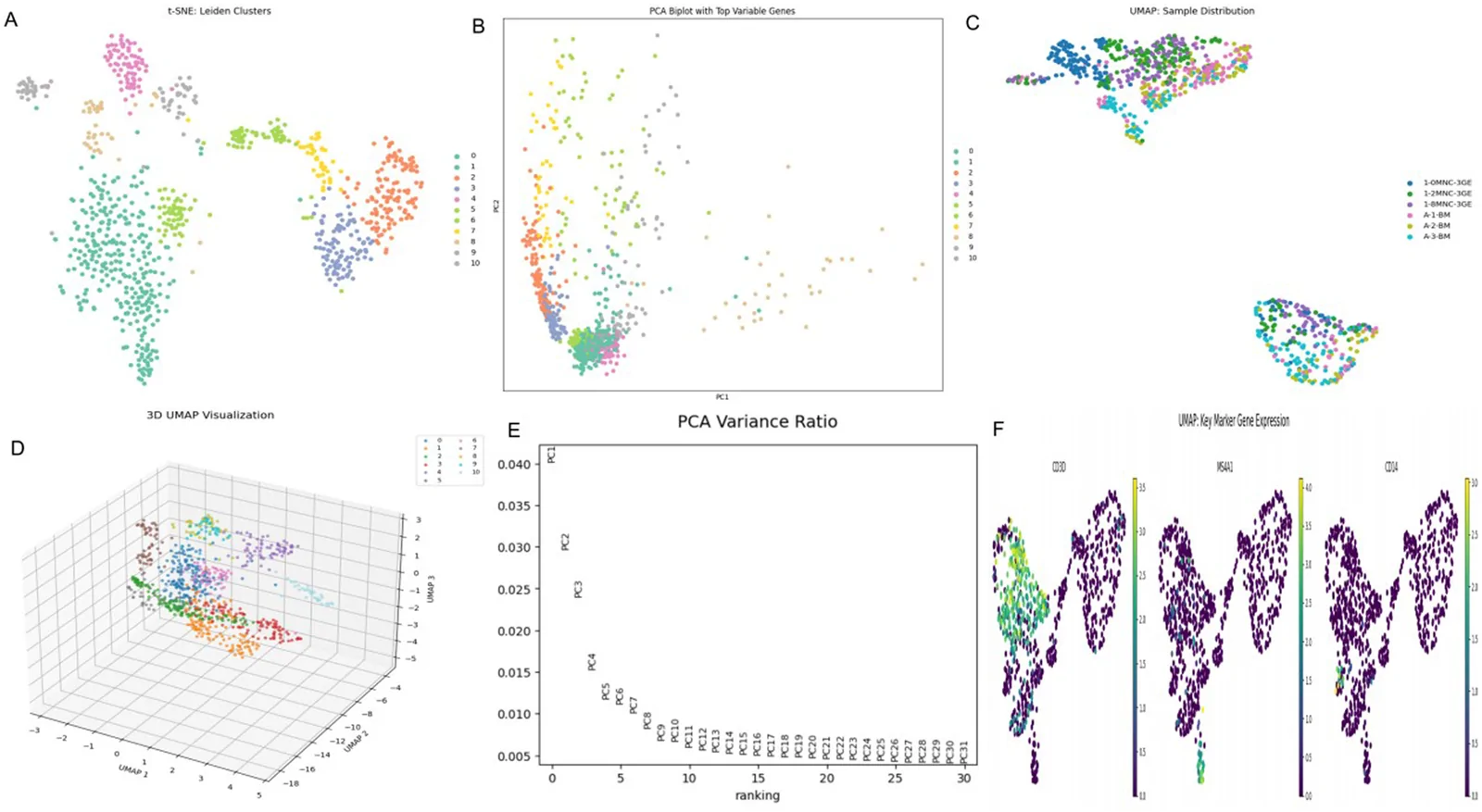
Dimensionality reduction of immune ecosystem states. **(A)** t-SNE of 11 Leiden clusters. **(B)** PCA biplot. **(C)** UMAP by sample origin. **(D)** Three-dimensional UMAP. **(E)** Variance explained by the first 30 PCs. **(F)** Representative marker-gene expression across timepoints.

### Transcriptional profiling of the MDS immune ecosystem composition

UMAP cell density analysis (Figure 3A) revealed regions of high cellular concentration corresponding to major immune ecosystem cell populations. Systematic marker gene expression profiling (Figure 3B) enabled precise ecosystem state cell type identification across 11 clusters, with CD3D marking T cell ecosystem states (clusters 0, 1), MS4A1 identifying B-lineage-associated states (clusters 2, 6), CD14 labeling monocyte/macrophage states, and CD4 defining helper T cell ecosystem states. Immune marker distribution analysis (Figure 3C) demonstrated that CD3D expression was prominent in clusters 0, 1, and 10, while MS4A1 expression was concentrated in B-lineage-associated clusters but remained relatively sparse in scRNA-seq data; B-cell ecosystem state annotation was therefore based on combined marker patterns rather than MS4A1 alone. The comprehensive marker gene expression matrix (Figure 3D) revealed ecosystem state-specific expression signatures with clear segregation of lineage-defining markers across cell types. Individual cluster analysis (Figure 3E) provided detailed characterization of gene expression patterns within each ecosystem state population. Hierarchical clustering analysis (Figure 3F) organized ecosystem state cell types based on transcriptional similarity, revealing relationships between immune cell subsets and identifying potential developmental trajectories within the MDS immune ecosystem.

**FIGURE 3:**
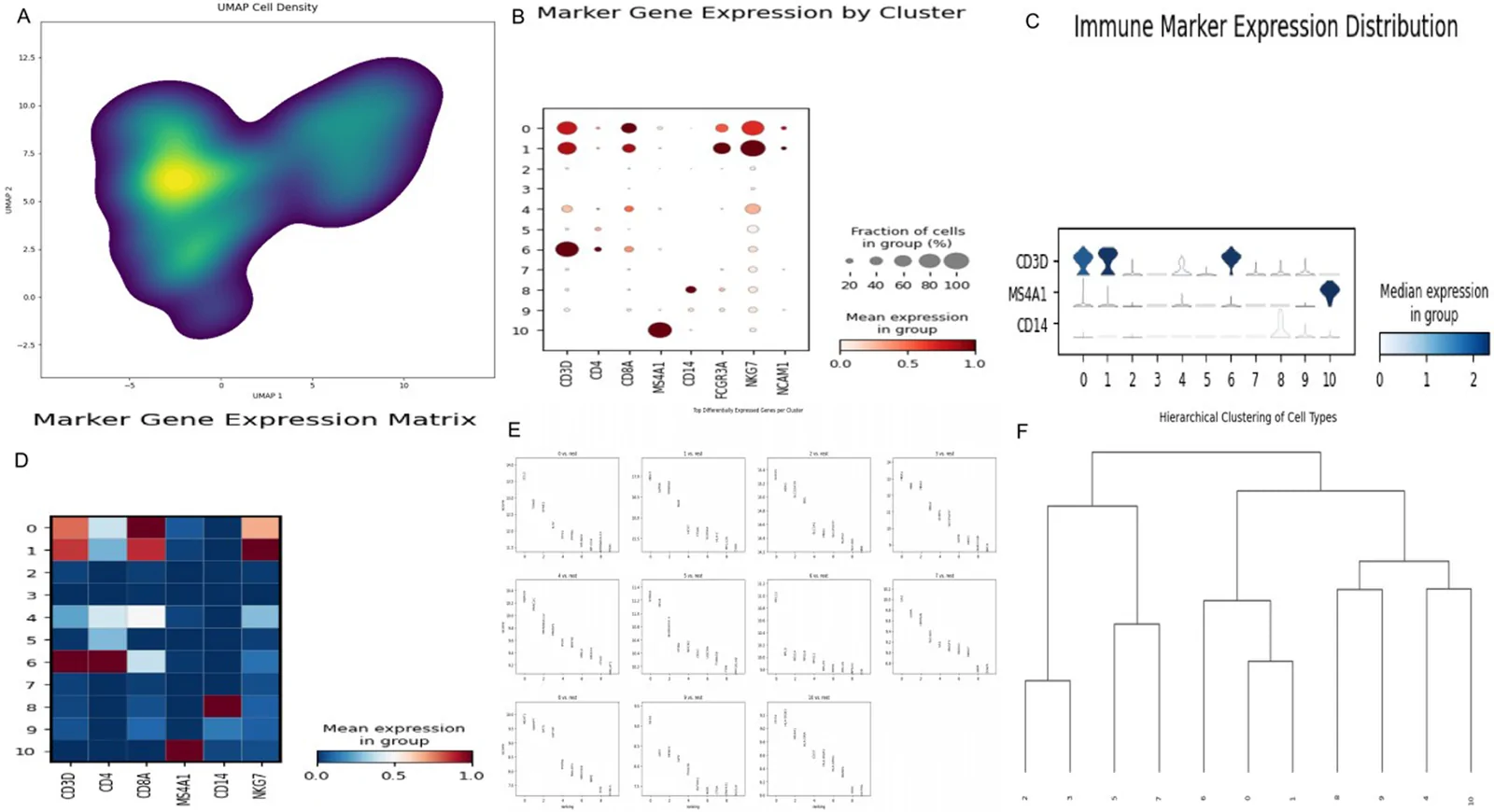
Transcriptional profiling of the MDS immune ecosystem. **(A)** UMAP cell density. **(B)** Marker-gene dot plot across 11 clusters. **(C)** Violin plots of CD3D, MS4A1, and CD14. **(D)** Marker-gene heatmap. **(E)** Cluster-specific expression profiles. **(F)** Hierarchical relationships among annotated states.

### Ecosystem state-specific immune checkpoint signatures and differential gene expression

Circular ecosystem state size distribution (Figure 4A) revealed that cluster 0 represented the largest population, followed by clusters 1, 2, and 10, indicating predominance of T cell and B-lineage ecosystem states in the MDS immune landscape. Sample-wise ecosystem state composition analysis (Figure 4B) demonstrated striking heterogeneity across samples, with control samples showing distinct patterns compared to MDS samples, particularly in the distribution of clusters 0, 1, and 2. Immune cell marker visualization (Figure 4C) confirmed spatial localization of CD3 (pan-T cell), CD79 (B cell), CD14 (monocyte), and MS4A1 (B cell) expression patterns across the UMAP ecosystem state landscape. Immune checkpoint genes (Figure 4D) including PDCD1 (PD-1), CTLA4, LAG3, and HAVCR2 (TIM-3) exhibited sparse but distinct expression patterns across ecosystem states, suggesting varying states of immune activation and exhaustion within the MDS immune ecosystem. Differential expression analysis comparing cluster 0 to all other ecosystem state clusters (Figure 4E) identified numerous significantly upregulated genes (Log2FC > 2, adjusted p-value <0.05), with the most significant changes reaching -Log10 (p-value) >30. MA plot analysis (Figure 4F) revealed a subset of highly expressed ecosystem state marker genes with substantial fold changes. These differentially expressed genes were interpreted as ecosystem state-associated or MDS-associated transcriptional features rather than strictly MDS-cell-specific markers.

**FIGURE 4:**
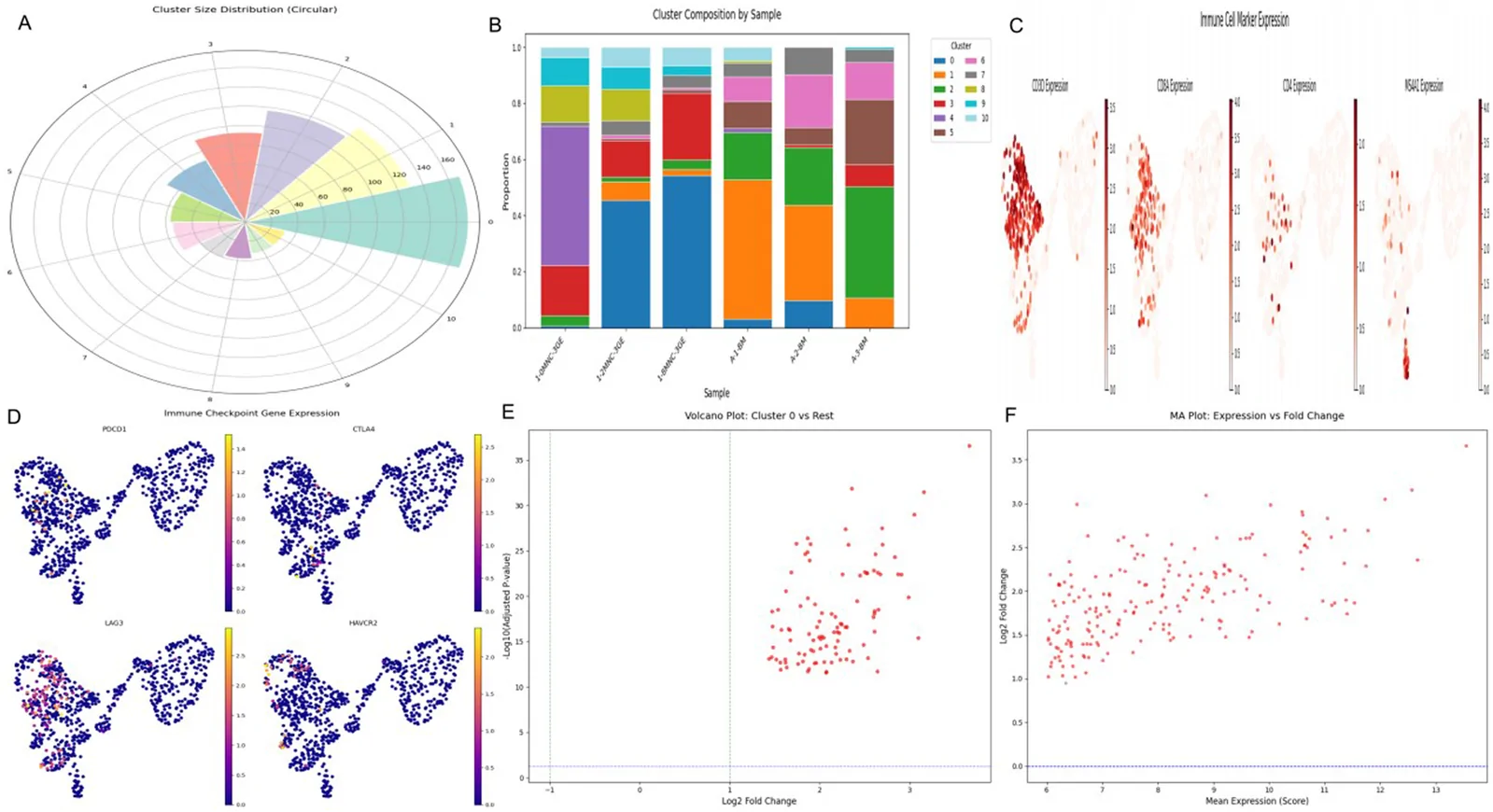
Ecosystem composition and checkpoint signatures. **(A)** Relative cluster sizes. **(B)** Sample-level cluster composition. **(C)** UMAP expression of CD3, CD79, CD14, and MS4A1. **(D)** Checkpoint expression (PDCD1, CTLA4, LAG3, HAVCR2) **(E)** Volcano plot for cluster 0 versus other clusters. **(F)** MA plot of differential expression.

### Cytokine and immunoregulatory program distribution across ecosystem states

Cytokine and chemokine expression analysis (Figure 5A) revealed limited expression across most ecosystem state clusters, with notable exception in cluster eight showing IL2, IL6, TNF, and CXCL8 transcript signals, suggesting this ecosystem state may harbor activated immune cells with inflammatory transcriptional programs. Protein-level and secretion-based assays would be required to confirm functional cytokine production. The comprehensive marker gene heatmap (Figure 5B) demonstrated distinct expression signatures across all ecosystem states, with clear separation of immune cell lineages and identification of ecosystem state-specific markers including HBB, MALAT1, B2M, ETS1, IRAK3, IL7R, TYMP, BTG2, IL32, RPL3, S100A9, HLA-DRA, MGST1, PPBP, AQP9, and related genes. CD3D expression distribution (Figure 5C) confirmed its restriction to specific ecosystem state clusters (primarily 0, 1, 5, and 10), validating T cell ecosystem state identity. MS4A1 (CD20) expression (Figure 5D) was highly specific to cluster 2, with minimal expression in other ecosystem state populations. Raincloud plot analysis (Figure 5E) revealed bimodal CD3A expression patterns across ecosystem states. Gene-gene correlation analysis (Figure 5F) between CD3D and MS4A1 demonstrated negative correlation (r = −0.04), consistent with their expression in distinct immune ecosystem state lineages. Because scRNA-seq measures transcript abundance, cytokine and chemokine ecosystem state expression patterns were interpreted as transcriptional evidence of cytokine-related programs rather than direct evidence of cytokine secretion.

**FIGURE 5:**
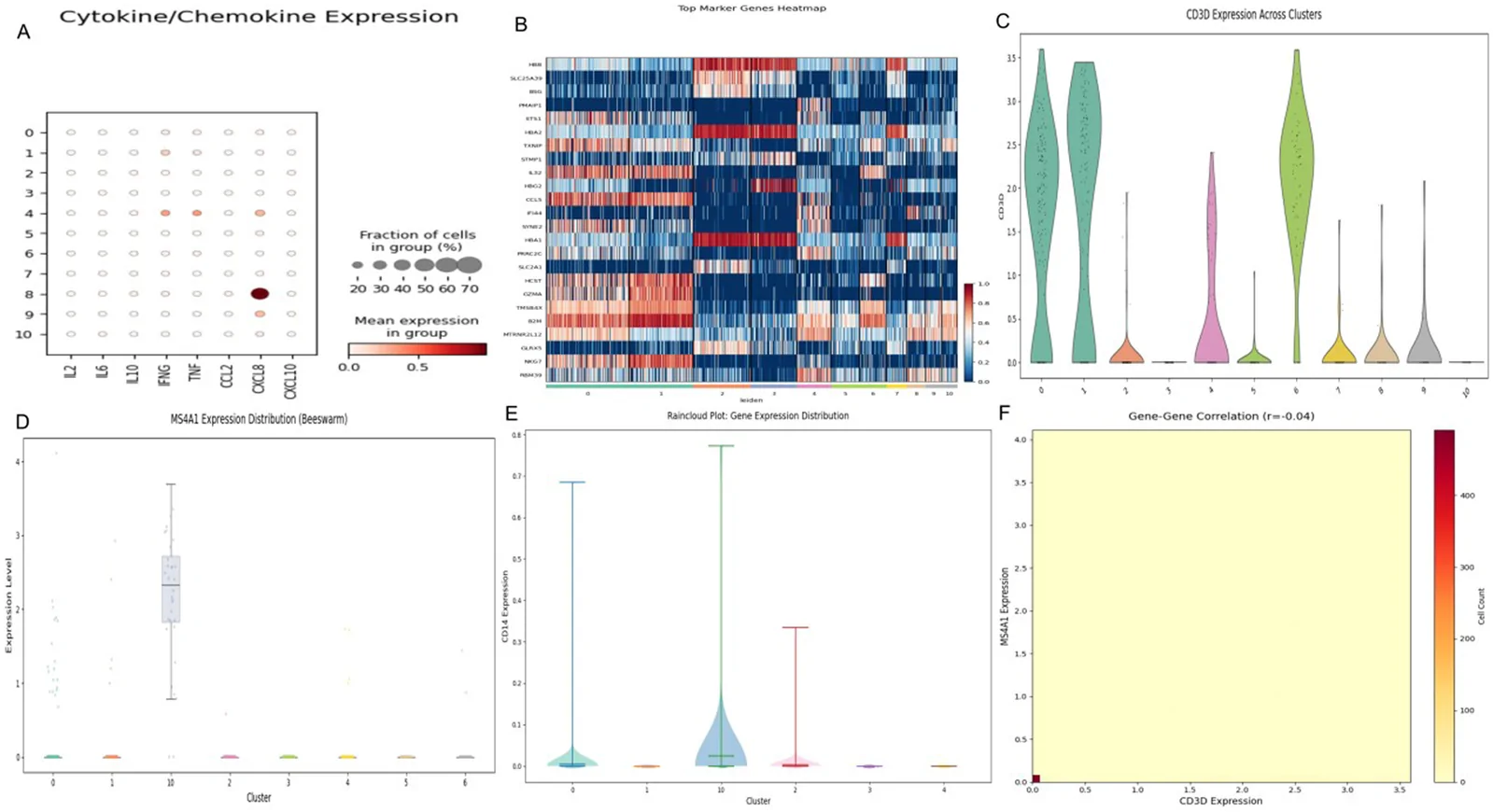
Cytokine and immunoregulatory expression across ecosystem states. **(A)** Cytokine/chemokine dot plot. **(B)** Marker-gene heatmap. **(C)** CD3D distribution. **(D)** MS4A1 expression. **(E)** CD3A raincloud plot. **(F)** CD3D–MS4A1 correlation.

### Pathway enrichment analysis reveals functional signatures of MDS ecosystem states

Gene expression track analysis across ecosystem states (Figure 6A) showed cluster-resolved expression of CD3D, CD4, CD8A, MS4A1, and CD14, separating lymphoid-associated and myeloid-associated ecosystem state marker patterns. The pathway enrichment lollipop plot (Figure 6B) ranked immune and signaling programs by enrichment score across ecosystem states, with T cell activation, interferon response, and cytokine signaling among the strongest signals, followed by apoptosis, MAPK signaling, cell proliferation, immune response, NF-κB pathway, antigen processing, and JAK-STAT pathway. The gene-pathway interaction network (Figure 6C) linked T cell-associated markers CD4 and CD3D, B-cell-associated MS4A1, NK-cell-associated NKG7, and myeloid-associated CD14 and FCGR3A, indicating lineage-associated regulatory modules operating within distinct MDS immune ecosystem states. The chord-style ecosystem state relationship network (Figure 6D) visualized inferred inter-ecosystem state relationships across C0–C10. The treemap (Figure 6E) quantified ecosystem state sizes, with cluster 0 (169 cells), cluster 1 (130 cells), cluster 2 (117 cells), cluster 3 (93 cells), cluster 4 (71 cells), cluster 5 (57 cells), cluster 6 (55 cells), cluster 7 (41 cells), cluster 8 (38 cells), cluster 9 (33 cells), and cluster 10 (32 cells). The waterfall plot (Figure 6F) displayed ranked gene-set enrichment scores with positive and negative values, supporting heterogeneous pathway activity across ecosystem state-associated gene sets.

**FIGURE 6:**
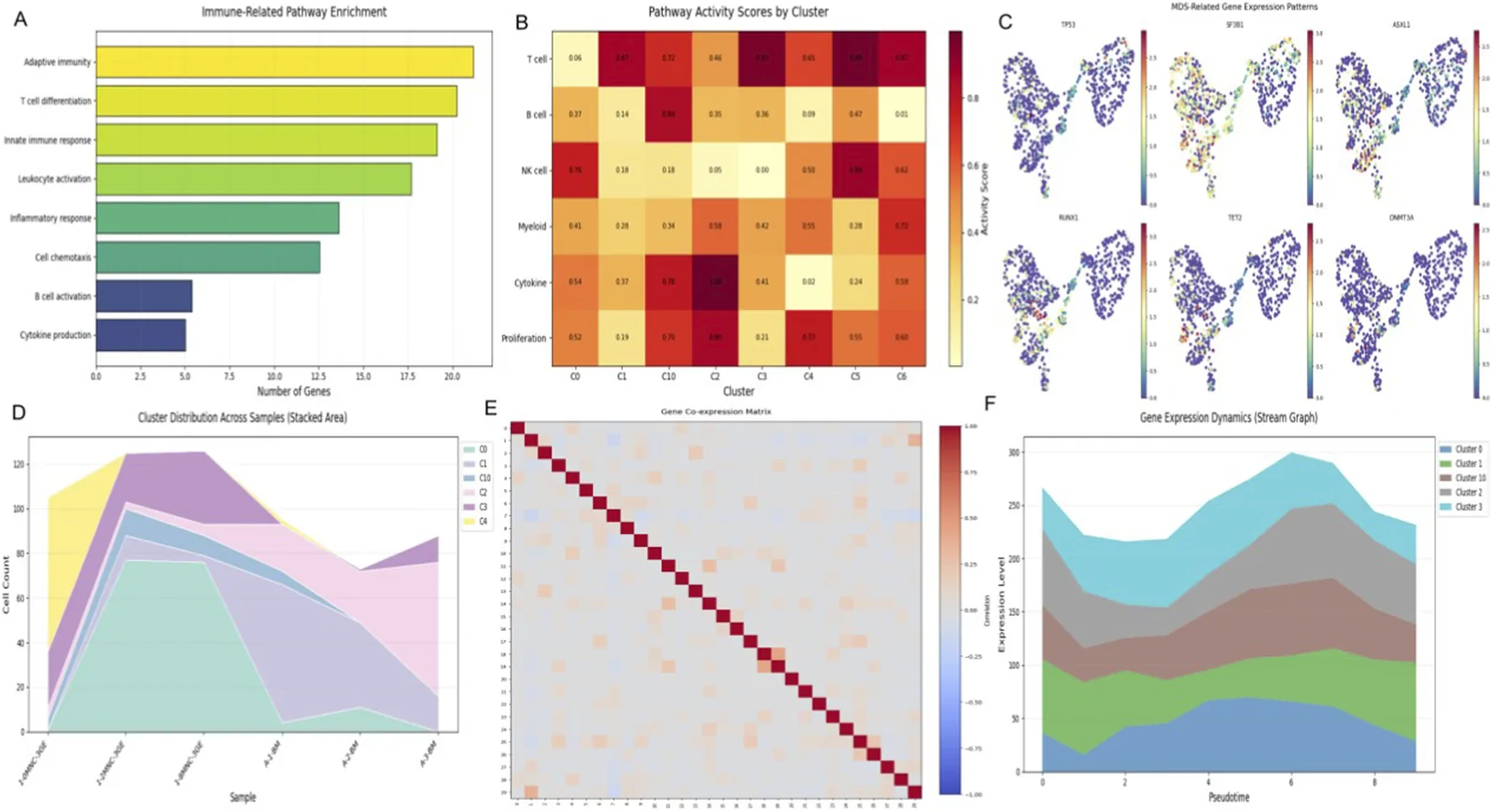
Immune module scores and MDS-associated gene expression. **(A)** Immune pathway enrichment. **(B)** Pathway activity heatmap. **(C)** UMAP expression of TP53, SF3B1, ASXL1, RUNX1, TET2, and DNMT3A. **(D)** Sample-level state distributions. **(E)** Gene co-expression matrix. **(F)** Ordered gene-expression stream graph.

### Immune module scoring and MDS-associated gene expression across ecosystem states

Immune-related pathway enrichment analysis (Figure 7A) ranked adaptive immunity as the largest gene-count category across MDS ecosystem states, followed by T cell differentiation, innate immune response, leukocyte activation, inflammatory response, cell chemotaxis, B cell activation, and cytokine production. Pathway activity score heatmap (Figure 7B) showed ecosystem state-specific activity patterns across annotated programs: T-cell program scores were high in C1, C10, C3, C5, and C6 but low in C0; B-cell activity was higher in C10 than in other clusters; cytokine activity was strongest in C2 and C10; NK-cell activity was high in C0, C5, and C6; and myeloid activity was most prominent in C6, C2, and C4. UMAP feature plots (Figure 7C) showed transcript-level expression of MDS-associated genes TP53, SF3B1, ASXL1, RUNX1, TET2, and DNMT3A across ecosystem states; these plots reflect gene-expression abundance and should not be interpreted as mutation status or clonal genotyping. The stacked area chart (Figure 7D) summarized sample-level ecosystem state distributions across six GSE241417 samples, showing sample-specific variation in ecosystem state composition. The gene co-expression matrix (Figure 7E) showed coordinated expression structure among ecosystem state-associated genes. The stream graph (Figure 7F) displayed ecosystem state-associated gene-expression dynamics along an ordered axis and should be interpreted as an exploratory ordered-expression visualization rather than evidence of formal developmental trajectory or temporal progression.

**FIGURE 7:**
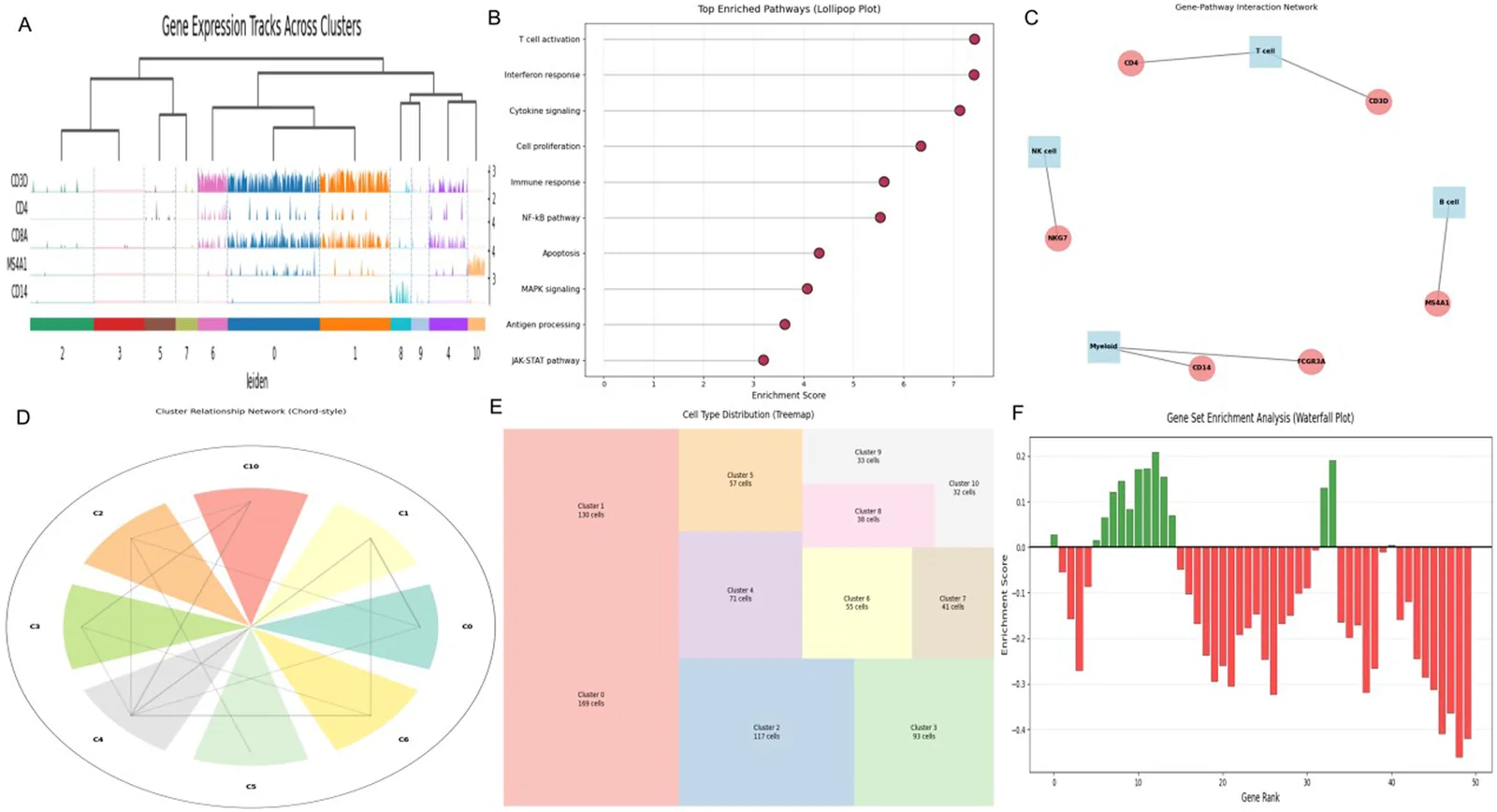
Pathway signatures across MDS immune ecosystem states. **(A)** Cluster-resolved gene-expression tracks. **(B)** Enriched pathways. **(C)** Gene-pathway network. **(D)** Inter-state relationship chord diagram. **(E)** Cluster-size treemap. **(F)** Ranked gene-set enrichment scores.

### Cell-cell communication networks define the MDS immune ecosystem architecture

Cell-cell interaction chord diagram (Figure 8A) visualized inferred communication interactions among immune ecosystem B cells, T cells, monocytes, NK cells, and DCs, with multiple connections among lymphoid and myeloid ecosystem populations. The ecosystem communication network (Figure 8B) showed T cells as a central node connected to DC, B-cell, monocyte, and NK-cell ecosystem nodes, with stronger communication links indicated by redder edges. The interaction strength matrix (Figure 8C) quantified directionality of ecosystem communication by source and target cell type, with the strongest displayed interactions including NK cells to DCs (0.98), B cells to T cells (0.96), NK cells to neutrophils (0.96), T cells to DCs (0.93), and monocytes to DCs (0.93). The ligand-receptor arc diagram (Figure 8D) summarized selected ligand/receptor and checkpoint-related ecosystem communication genes, including CD3D, CD28, CD80, CD86, PDCD1, CD274, IFNG, and IFNGR1. The multi-layer annotated heatmap (Figure 8E) integrated ecosystem state cluster labels, sample annotations, and expression values. The comprehensive ecosystem analysis dashboard (Figure 8F) summarized UMAP clusters, cluster counts, QC relationships, feature plots for CD3D, MS4A1, and CD14, sample composition, PCA variance, and dataset summary metrics: total cells = 1,019, total genes = 19,819, ecosystem state clusters = 11, samples = 6, mean genes/cell = 3,610, max UMI/cell = 8,076, and mean mitochondrial percentage = 0.9%. Selected ligand/receptor and checkpoint-related ecosystem communication genes shown in Figure 8D should be interpreted as components of inferred communication axes rather than experimentally validated ligand-receptor pairs.

**FIGURE 8:**
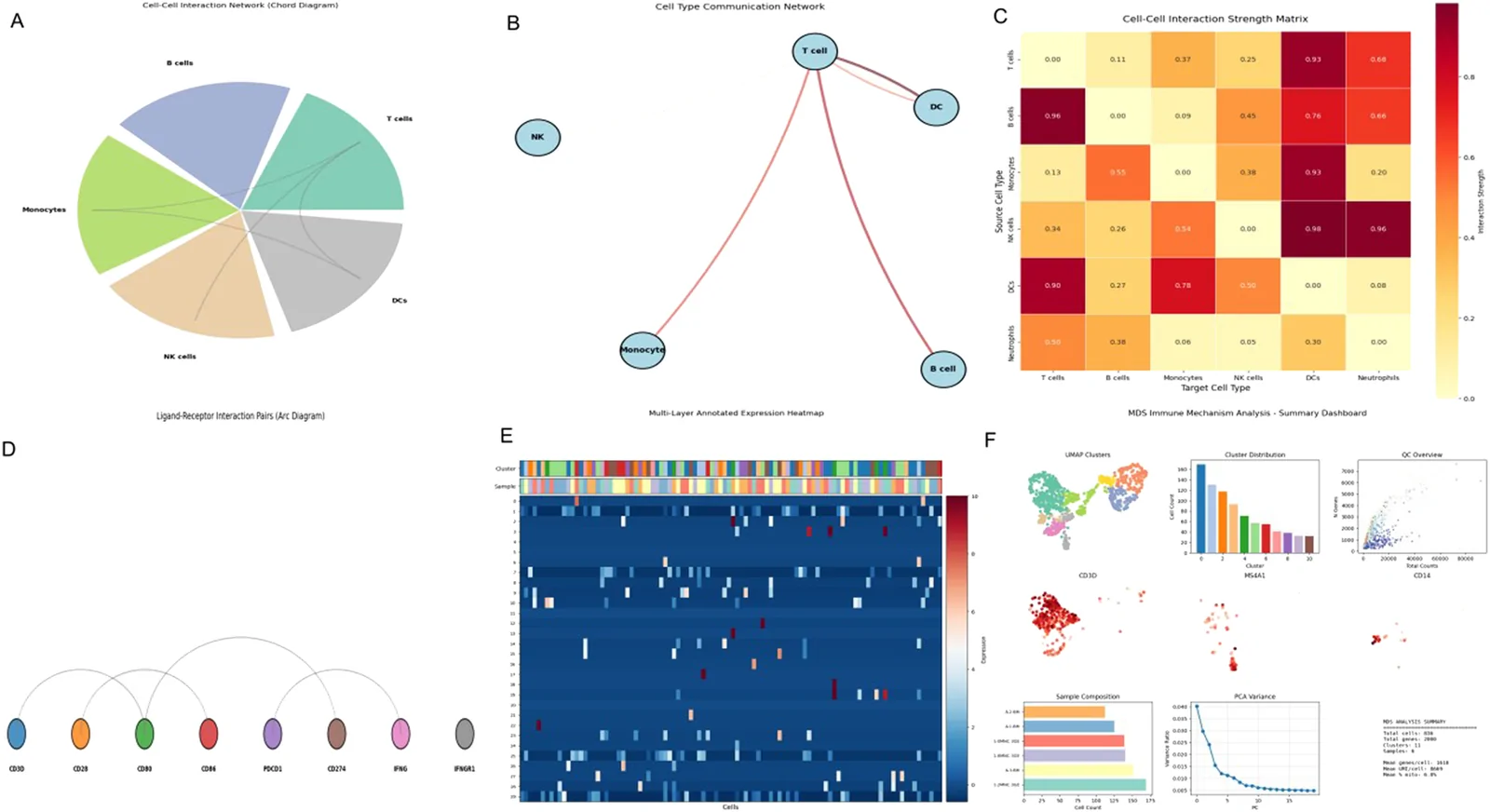
Inferred cell–cell communication in the MDS immune ecosystem. **(A)** Cell-type interaction chord diagram. **(B)** Directional communication network. **(C)** Source–target interaction matrix. **(D)** Selected ligand/receptor and checkpoint genes. **(E)** Integrated annotation heatmap. **(F)** Summary dashboard of clustering, QC, composition, and dataset metrics.

### Disease-associated ecosystem remodeling between MDS and healthy bone marrow

To place the MDS immune ecosystem in a broader disease-comparison context and further refine the inferred immune ecosystem state interactions, we performed a descriptive integration analysis using GSE241417 as the MDS cohort and GSE120221 as an external healthy bone marrow reference. Split UMAP visualization showed that healthy reference and MDS cells occupied different low-dimensional ecosystem regions, indicating differences in both immune ecosystem composition and transcriptional-state organization (Figure 9A). Sample-level ecosystem cell-type composition further revealed substantial inter-sample heterogeneity, with redistribution of T cells, NK cells, and other immune populations across MDS ecosystem state samples (Figure 9B). A descriptive ecosystem state proportion comparison suggested relative enrichment of T/NK-related populations in MDS and redistribution of other marrow cell compartments (Figure 9C). Cell-type-resolved ecosystem state expression analysis indicated that disease-associated changes were not limited to abundance shifts but also involved altered marker, cytotoxic, checkpoint, inflammatory, and antigen-presentation programs within specific ecosystem cell types (Figure 9D). Functional module scoring supported changes in inflammatory response, interferon response, T-cell activation, exhaustion-like, and antigen-presentation programs across MDS ecosystem states (Figure 9E). Sample-level PCA based on immune ecosystem composition showed a separation trend between MDS and healthy reference samples (Figure 9F). Because the healthy comparator was derived from an external dataset, these findings were interpreted as descriptive and hypothesis-generating rather than definitive population-level conclusions about MDS ecosystem remodeling.

**FIGURE 9:**
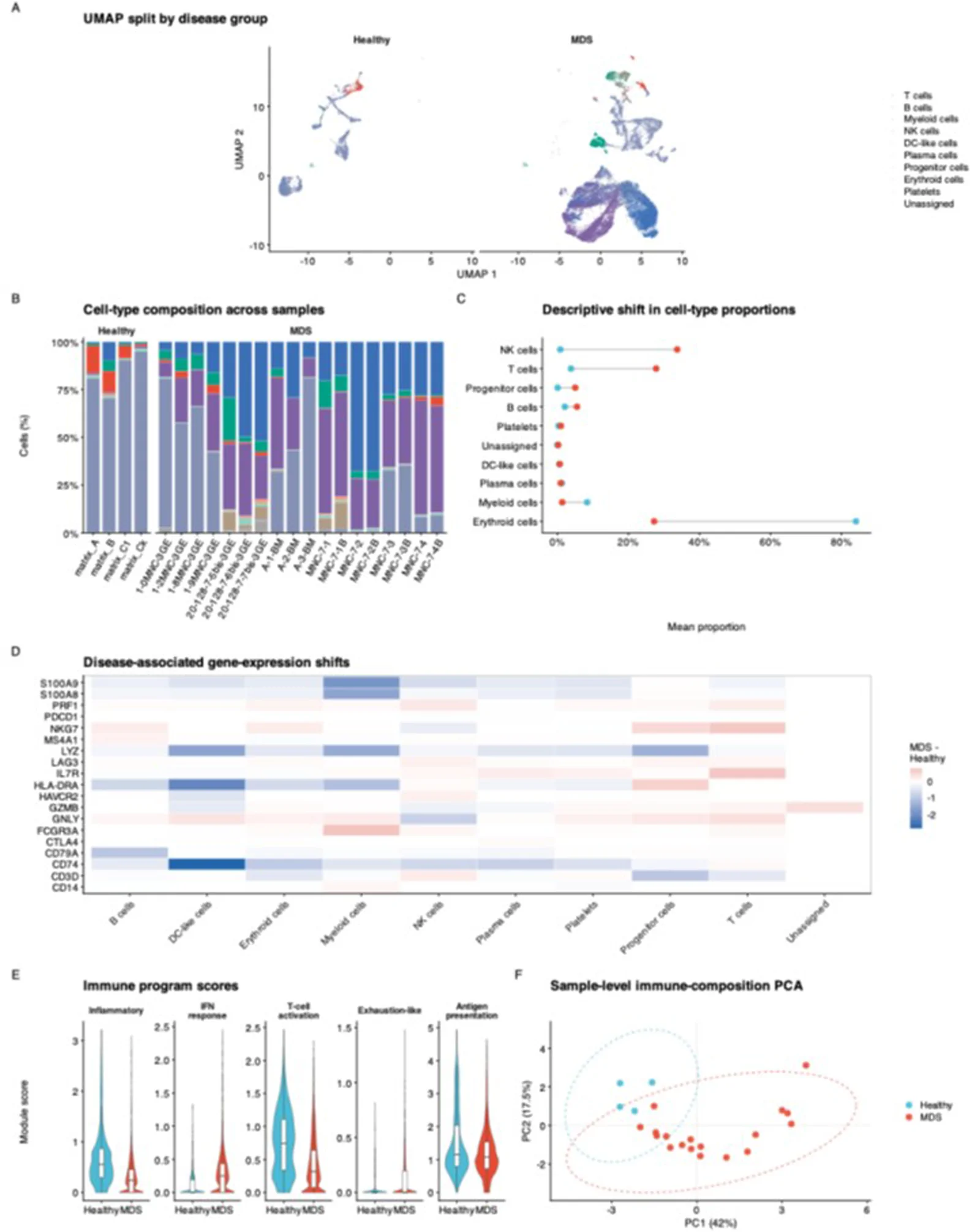
Descriptive MDS–healthy bone marrow comparison. **(A)** Split UMAP by condition. **(B)** Sample-level cell-type composition. **(C)** Descriptive cell-type proportion comparison. **(D)** Cell-type-resolved immune-gene heatmap. **(E)** Functional module scores. **(F)** Sample-level PCA. Healthy cells were obtained from an external dataset; comparisons are descriptive.

### T/NK-cell ecosystem state remodeling in MDS

To further define lymphoid ecosystem state dysfunction, T/NK cells were extracted and reclustered to characterize disease-associated lymphoid functional ecosystem states. The T/NK ecosystem state UMAP resolved CD4-like T cells, CD8 cytotoxic T cells, exhaustion-like T cells, Treg-like cells, NK/NKT-like cells, and intermediate states, with partial redistribution of these states in the MDS ecosystem (Figure 10A). Ecosystem state proportion analysis suggested that CD8 cytotoxic T cells, exhaustion-like T cells, and T/NK intermediate states increased in MDS, whereas CD4-like T cells and NK/NKT-like cells were relatively reduced (Figure 10B). Marker-gene analysis validated the ecosystem state annotations: CD8 cytotoxic states expressed NKG7, GNLY, GZMB, and PRF1; exhaustion-like states expressed PDCD1, LAG3, and TIGIT; and Treg-like states expressed FOXP3, IL2RA, IKZF2, and CTLA4 (Figure 10C). Functional scoring further separated cytotoxic, activated, exhaustion-like, and Treg-like programs across T/NK ecosystem states (Figure 10D). Checkpoint co-expression analysis showed enhanced PDCD1, CTLA4, LAG3, HAVCR2, TIGIT, CD274, and PDCD1LG2 patterns in MDS-associated T/NK ecosystem states, particularly in exhaustion-like and selected cytotoxic or NK-associated states (Figure 10E). Mapping cells along a cytotoxicity-exhaustion axis indicated that some MDS T/NK ecosystem states simultaneously retained cytotoxic features while acquiring exhaustion-like programs, suggesting a sustained-activation and functional-imbalance phenotype characteristic of immune ecosystem dysfunction in MDS (Figure 10F).

**FIGURE 10:**
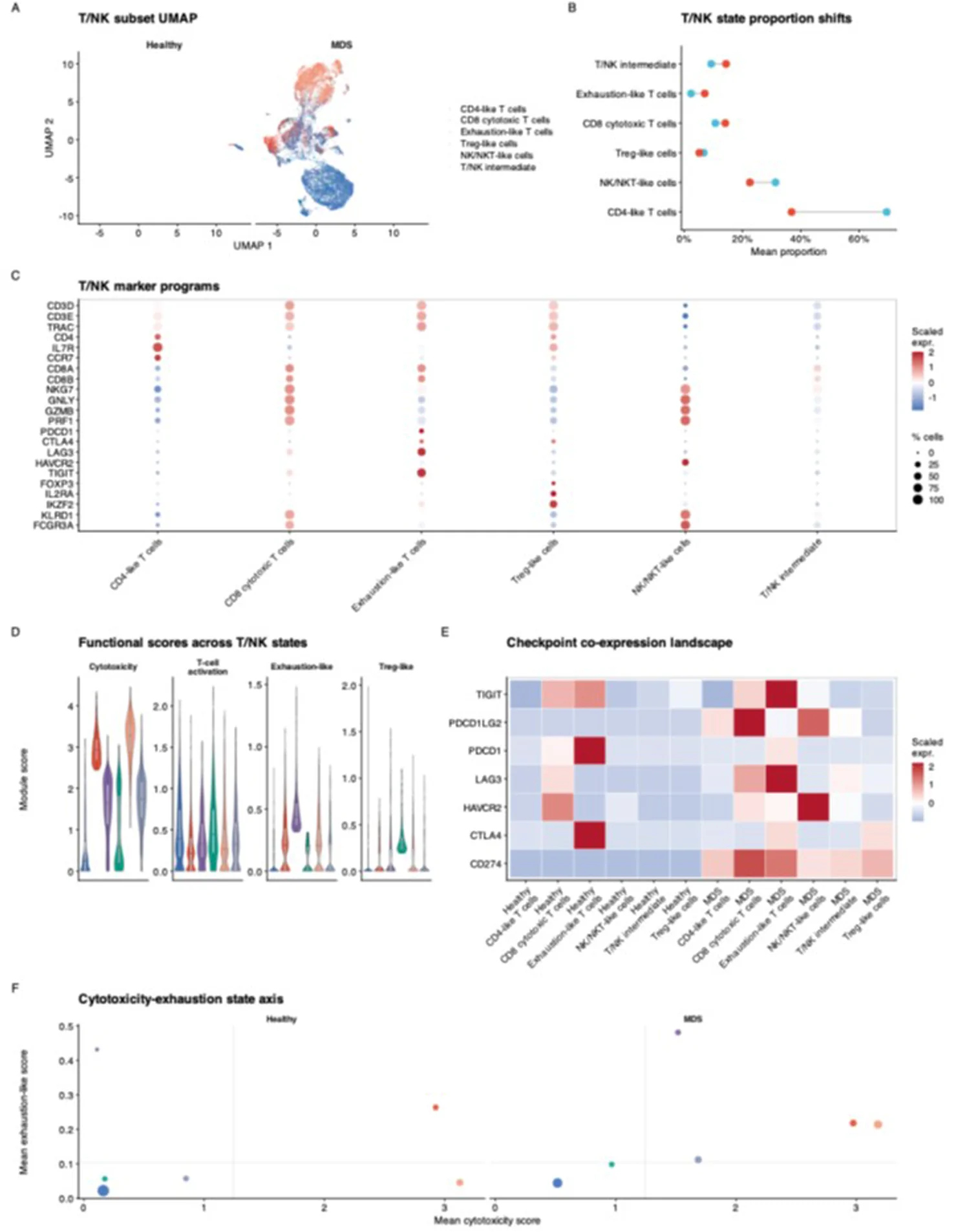
T/NK-cell state remodeling in MDS. **(A)** UMAP of T/NK subpopulations. **(B)** Descriptive state-proportion shifts. **(C)** Marker-gene dot plot **(D)** Cytotoxicity, activation, exhaustion-like, and Treg-like module scores. **(E)** Checkpoint co-expression patterns. **(F)** Cytotoxicity–exhaustion functional axis.

### Single-cell localization of immunosuppressive programs across ecosystem states

Because subsequent functional validation focused on IL10, TGFB1, CD274, and FOXP3, we mapped these immunoregulatory genes at single-cell ecosystem state resolution to provide transcriptional context for the cell-line experiments. UMAP feature maps showed that TGFB1 was broadly detectable across multiple immune ecosystem state regions, whereas IL10, CD274, and FOXP3 displayed sparser, ecosystem state-dependent expression patterns (Figure 11A). Cell-type-resolved ecosystem state expression further showed that cytokine and checkpoint genes were distributed across multiple immune ecosystem compartments rather than being restricted to a single source population (Figure 11B). Correlation analysis demonstrated that the relationship between CD274 and IL10, TGFB1, or FOXP3 varied by ecosystem state cell type, suggesting that CD274-associated regulation may be context dependent within the MDS immune ecosystem (Figure 11C). Heatmap analysis of immunosuppressive, inflammatory, antigen-presentation, and checkpoint genes showed that MDS-associated immune ecosystem states can combine inflammatory activation with inhibitory checkpoint programs simultaneously (Figure 11D). When cells were stratified by CD274 expression, CD274-high cells showed higher TGFB, checkpoint, and immunoregulatory module scores than CD274-low cells, representing an association with CD274 expression rather than evidence of direct regulation (Figure 11E). CD274-high cells represented a low-frequency but multi-source ecosystem population detectable across T cells, NK cells, B cells, and other immune cell types (Figure 11F). These findings provide single-cell ecosystem context for the subsequent functional analysis of CD274-dependent regulation of IL-10 and FOXP3.

**FIGURE 11:**
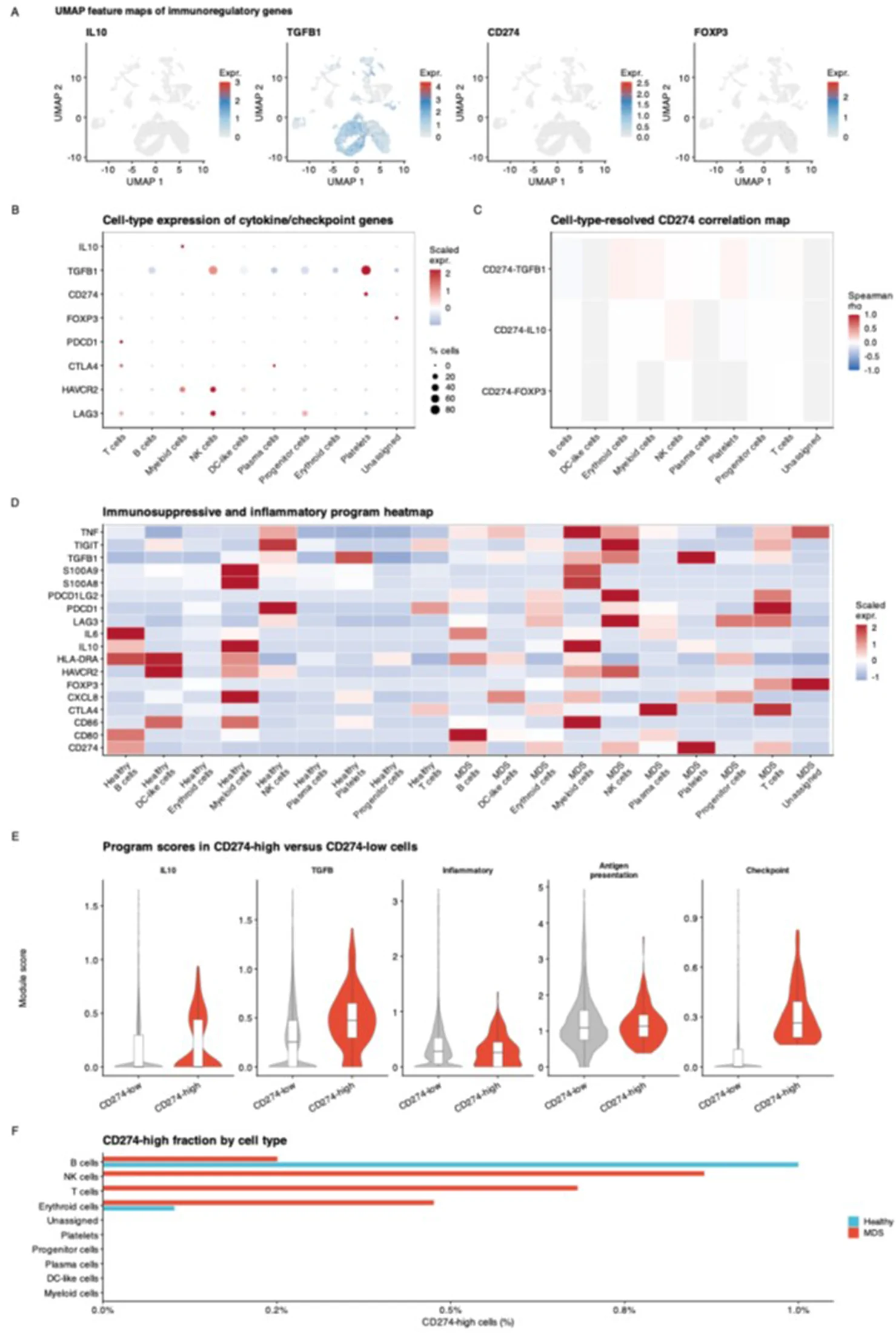
Single-cell localization of immunosuppressive programs. **(A)** UMAP expression of IL10, TGFB1, CD274, and FOXP3. **(B)** Cell-type expression of cytokine and checkpoint genes. **(C)** Cell-type-resolved correlations with CD274. **(D)** Immunoregulatory gene heatmap. **(E)** Module scores in CD274-high versus CD274-low cells. **(F)** Distribution of CD274-high cells across cell types.

### CD274-centered communication network coordinates immunosuppressive ecosystem signaling in MDS

Finally, we focused the ligand-receptor analysis on CD274 and other immunosuppressive communication axes to characterize the CD274-centered communication network shaping the MDS immune ecosystem. Comparison of estimated communication strength indicated that TGFB1-TGFBR1/2, PDCD1LG2-PDCD1, CD274-PDCD1, CD80/CD86-CTLA4, and LGALS9-HAVCR2 axes showed higher estimated scores in MDS than in the external healthy reference in this descriptive analysis (Figure 12A). CD274-centered ecosystem communication mapping showed that multiple CD274-expressing sender ecosystem populations were paired with PDCD1-positive T/NK receiver states in the inferred communication map, indicating that CD274-PDCD1 ecosystem communication may be multi-source rather than restricted to a single cell type (Figure 12B). A focused ligand-receptor ecosystem landscape showed that CD274-PDCD1 coexisted with TGFB, LGALS9-HAVCR2, IL10, IFNG, CD80/CD86-CD28, and CD80/CD86-CTLA4 signaling, supporting a multi-axis immunosuppressive communication ecosystem model (Figure 12C). Sender-ligand-receptor-receiver flow analysis highlighted myeloid, NK, and T-cell sender ecosystem populations connected to T/NK and myeloid receiver populations through TGFB1, LGALS9, and related ligands (Figure 12D). Receiver ecosystem state gene-expression analysis showed that MDS T/NK states simultaneously contained cytotoxic genes and inhibitory or regulatory programs (Figure 12E). The CD274-PDCD1 sender-receiver matrix further localized the main cellular sources and potential receiver populations of CD274-associated ecosystem communication (Figure 12F). Collectively, these computational analyses suggest that CD274-PDCD1 may coexist with TGFB1-TGFBR, LGALS9-HAVCR2, and other inferred axes within a multi-layered immunosuppressive network; functional cooperation remains to be experimentally established.

**FIGURE 12:**
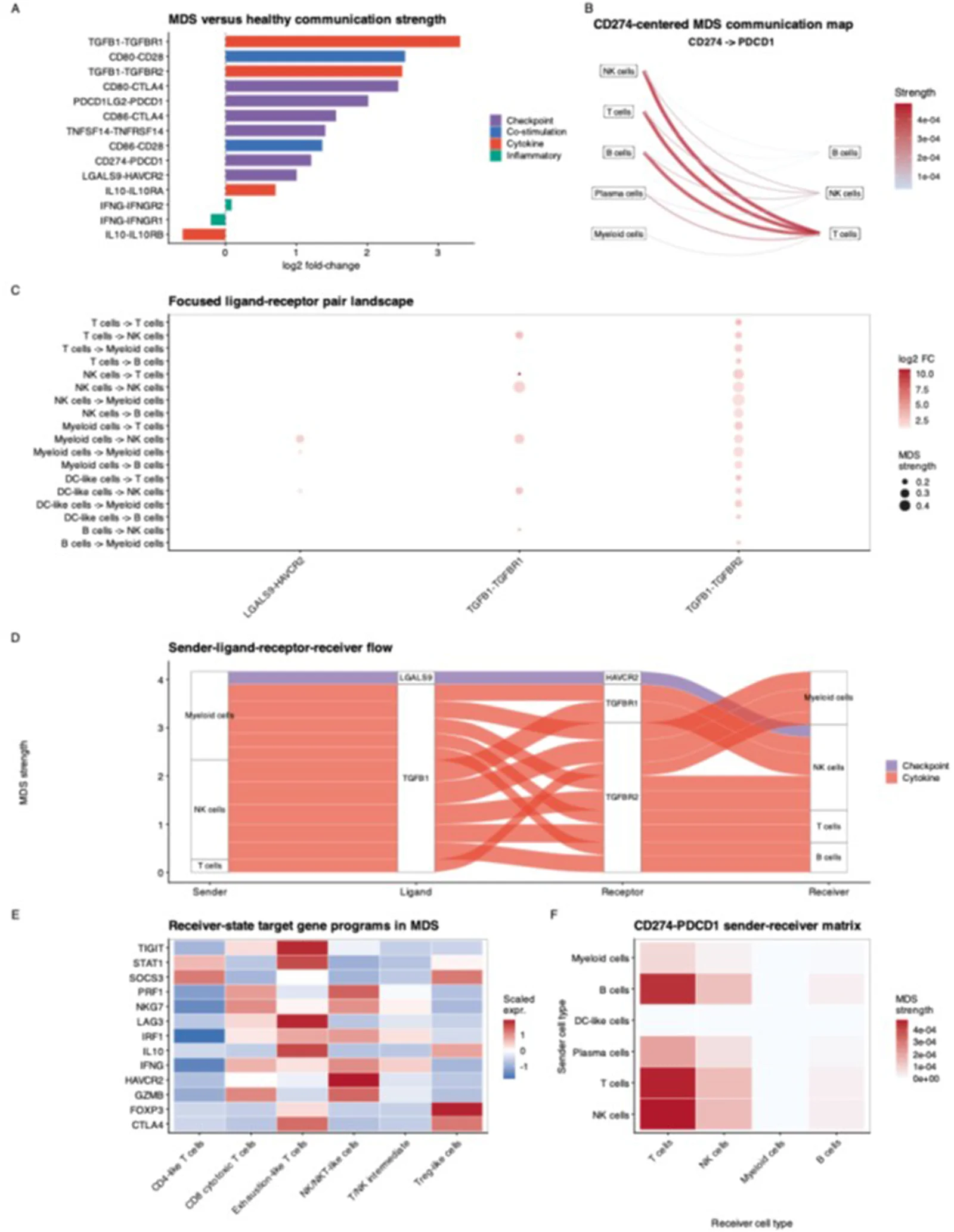
Computationally inferred CD274-centered communication network. **(A)** Descriptive MDS–healthy comparison of selected ligand–receptor scores. **(B)** CD274–PDCD1 sender–receiver map. **(C)** Selected immunoregulatory ligand–receptor axes. **(D)** Sender–ligand–receptor–receiver flow. **(E)** Receiver-state gene-expression heatmap. **(F)** CD274–PDCD1 sender–receiver matrix.

### RT-PCR validation of key immune regulatory genes in MDS cell lines

Based on the single-cell localization of IL10, TGFB1, CD274, and FOXP3 across MDS immune ecosystem states and the CD274-centered ecosystem communication analysis, we next performed RT-PCR validation in MDS-derived cell-line models. SKM-1 and MUTZ-1 cells were used as reductionist models to evaluate whether key ecosystem immune regulatory genes were expressed in MDS-related cellular contexts and whether CD274 perturbation could modulate IL-10 and FOXP3-related expression. Quantitative real-time PCR analysis of four critical immune regulatory genes (IL10, TGFB1, CD274, and FOXP3) in two MDS-derived cell lines (SKM-1 and MUTZ-1) was performed, compared to normal CD34^+^ control cells. Figure 13 shows relative mRNA expression levels normalized to GAPDH, revealing significantly elevated IL10 expression in both SKM-1 (3.2-fold, p < 0.01) and MUTZ-1 (2.8-fold, p < 0.01) compared to controls, consistent with the immunoregulatory transcriptional programs observed in the ecosystem state scRNA-seq analysis. TGFB1 expression was markedly upregulated in SKM-1 (4.1-fold, p < 0.001) and moderately increased in MUTZ-1 (2.3-fold, p < 0.05). CD274 (PD-L1) showed substantial elevation in SKM-1 (5.7-fold, p < 0.001) and MUTZ-1 (3.9-fold, p < 0.001), supporting the presence of checkpoint-associated ecosystem transcript expression. FOXP3 showed ecosystem model-specific patterns, with significant upregulation in SKM-1 (2.6-fold, p < 0.01) but minimal change in MUTZ-1 (1.2-fold, p = 0.34), suggesting heterogeneity in FOXP3-related ecosystem regulatory programs across MDS-derived cell-line models.

**FIGURE 13:**
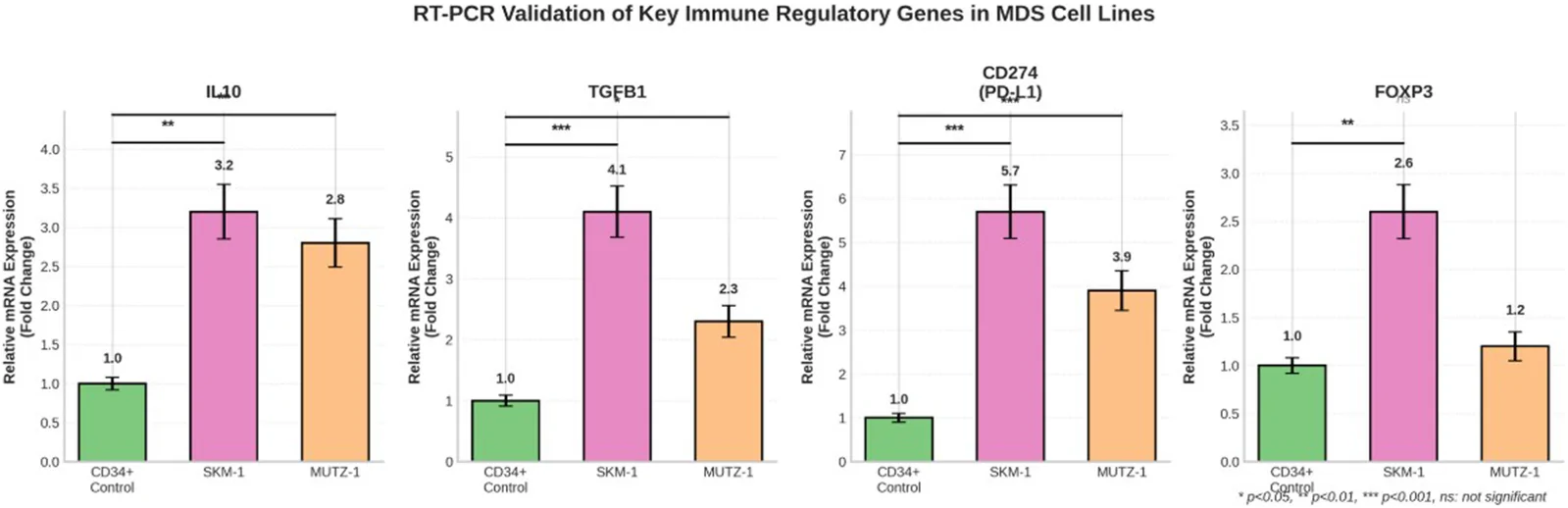
RT-PCR validation of immune regulatory genes. Relative mRNA expression of IL10, TGFB1, CD274 (PD-L1), and FOXP3 in SKM-1 and MUTZ-1 cells compared with CD34+ controls.

### CD274 positively regulates IL-10 expression in MDS-Derived cell-line ecosystem models

Immunofluorescence staining and quantitative analysis revealed that CD274 positively regulates IL-10 expression in both SKM-1 and MUTZ-1 MDS-derived cell lines. In SKM-1 cells, knockdown of CD274 using siRNA (si-CD274) significantly reduced IL-10 immunofluorescence signal (red) compared to control cells, while CD274 overexpression (CD274-OE) markedly enhanced IL-10 staining intensity. β-Tubulin (green) was used as a cytoskeletal marker, and DAPI (blue) counterstaining demonstrated comparable cell density across all treatment groups. Similar results were observed in MUTZ-1 cells, where si-CD274 treatment substantially decreased IL-10 signal intensity, whereas CD274-OE resulted in prominent IL-10 upregulation. Quantitative analysis confirmed that si-CD274 treatment reduced relative IL-10 expression to approximately 0.3-fold in SKM-1 cells and 1.0-fold in MUTZ-1 cells compared to controls (p < 0.001). CD274 overexpression increased IL-10 expression to approximately 2.7-fold in SKM-1 cells and 3.0-fold in MUTZ-1 cells (p < 0.001). These findings demonstrate that CD274 positively regulates IL-10 expression in MDS-derived cell-line ecosystem models, supporting a potential mechanistic link between CD274 signaling and immunosuppressive cytokine programs within the MDS immune ecosystem. Quantitative immunofluorescence analysis showed CD274-associated changes in IL-10 signal intensity under matched imaging conditions (Figures 14A–C).

**FIGURE 14:**
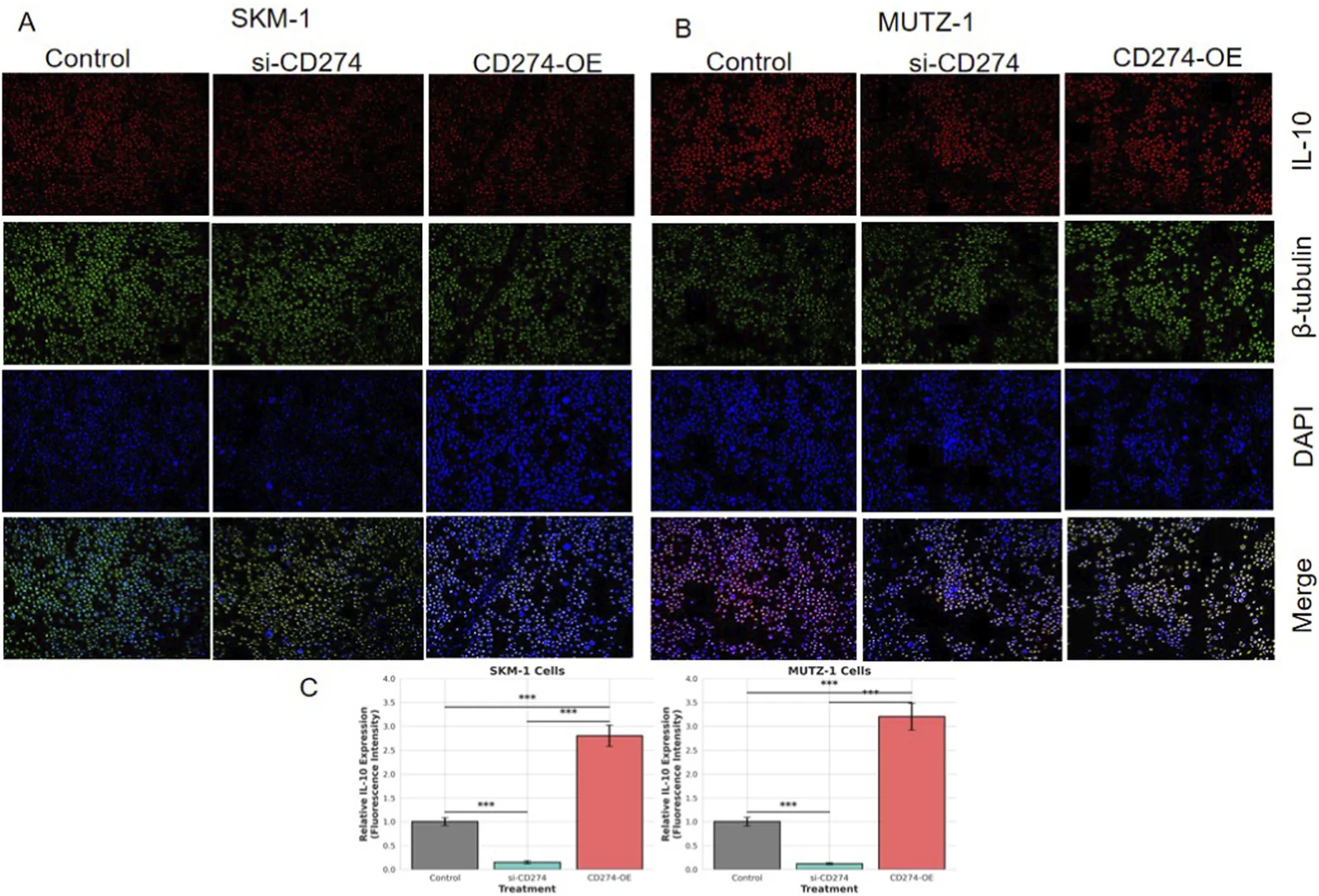
CD274 perturbation and IL-10 expression in SKM-1 and MUTZ-1 cells **(A,B)** Representative IL-10 (red), β-tubulin (green), and DAPI (blue) immunofluorescence after control, si-CD274, or CD274-OE treatment. **(C)** Relative IL-10 signal normalized to control. Mean ± SEM; ***p < 0.001.

### CD274 negatively regulates FOXP3 expression in MDS-Derived cell-line ecosystem models

To investigate the regulatory relationship between CD274 and FOXP3 within the MDS immune ecosystem regulatory program, we performed immunofluorescence staining in SKM-1 and MUTZ-1 cells under different CD274 modulation conditions. In SKM-1 cells, CD274 knockdown (si-CD274) significantly increased FOXP3-positive cells from 15.0% ± 2.5% to 45.0% ± 5.2% (p < 0.001), while CD274 overexpression (CD274-OE) maintained baseline levels (12.0% ± 2.1%). MUTZ-1 cells displayed higher baseline FOXP3 expression (52.0% ± 4.8%), which further increased to 78.0% ± 6.5% (p < 0.01) upon CD274 knockdown. CD274 overexpression in MUTZ-1 cells sustained high FOXP3 expression (68.0% ± 5.3%), suggesting additional regulatory mechanisms in this ecosystem cell-line model. These data demonstrate that CD274 negatively regulates FOXP3 expression in MDS-derived cell-line ecosystem models, with more pronounced effects in cells with lower baseline FOXP3 levels (Figures 15A–C). This result should be interpreted as a cell-line-based ecosystem regulatory observation rather than direct evidence of Treg behavior in primary MDS bone marrow.

**FIGURE 15:**
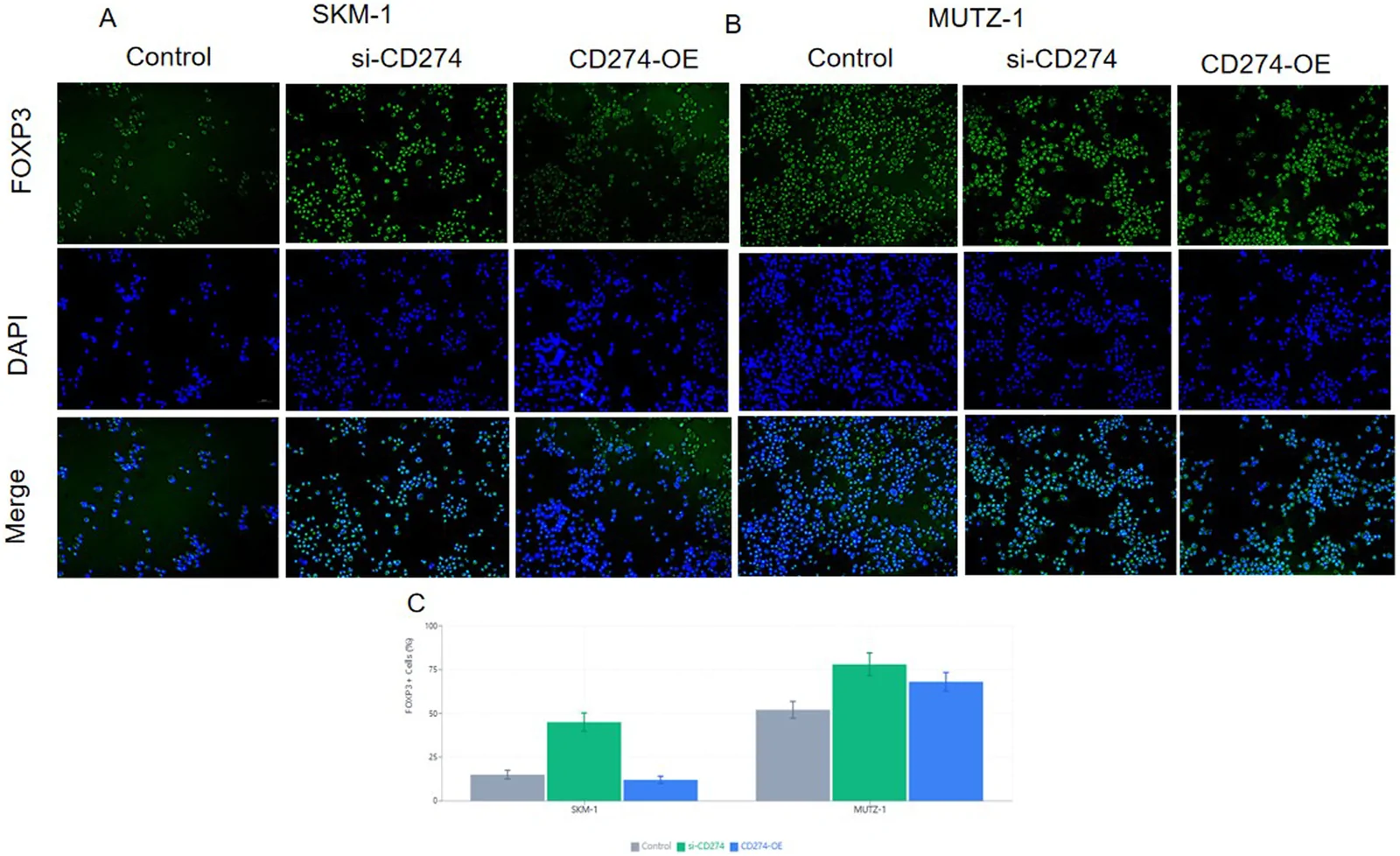
CD274 perturbation and cell-line-dependent FOXP3 changes. **(A,B)** Representative FOXP3 (green) and DAPI (blue) staining in SKM-1 and MUTZ-1 cells after control, si-CD274, or CD274-OE treatment. **(C)** Percentage of FOXP3-positive cells.

## Discussion

This study provides a computational single-cell transcriptomic characterization of the immune ecosystem states in myelodysplastic syndromes, revealing disease-associated remodeling of immune ecosystem composition, checkpoint molecule expression patterns, and intercellular communication networks that collectively define the MDS immunosuppressive ecosystem. Our findings extend the current understanding of immune dysregulation as a central driver of MDS pathogenesis by providing ecosystem-state-level resolution of these complex immunological processes, and identify CD274-associated transcriptional and communication patterns that warrant further mechanistic investigation.

In the expanded disease-control ecosystem analysis, integration of the MDS cohort with an external healthy bone marrow reference placed these immune alterations in a broader comparative ecosystem context. Because the healthy reference was generated independently, residual differences in platform, sample processing, sequencing depth, donor composition, and batch structure may remain despite harmonization; therefore, the analysis suggested that MDS bone marrow is characterized not only by changes in immune ecosystem cell composition but also by coordinated remodeling of T/NK-cell functional ecosystem states, inflammatory programs, checkpoint activity, and antigen-presentation signatures. The redistribution of cytotoxic, exhaustion-like, and regulatory T/NK ecosystem states supports a model in which persistent immune activation coexists with progressive immune dysfunction at the ecosystem level. Single-cell localization of IL10, TGFB1, CD274, and FOXP3, together with CD274-centered ligand-receptor ecosystem analysis, suggests that immune suppression in MDS is unlikely to be driven by a single cell type or pathway. Instead, CD274-PDCD1 signaling may cooperate with TGFB, IL10, LGALS9-HAVCR2, and costimulatory/inhibitory axes to shape a multi-layered immunosuppressive bone marrow ecosystem.

One of the most significant ecosystem state findings is the substantial upregulation of FOXP3 in MDS cell lines, confirmed by RT-PCR validation showing a 2.6-fold increase in SKM-1 cells. This observation should not be interpreted as evidence that MDS-derived cell lines represent Tregs; rather, it indicates that selected Treg-associated regulatory transcripts can be expressed in MDS-derived cellular models, consistent with the ecosystem state patterns observed in the scRNA-seq analysis. This finding aligns with previous reports demonstrating that expansion of CD4^+^CD25highFoxp3+ regulatory T cells is a characteristic feature of higher-risk MDS (Kordasti et al., 2007). Kordasti and colleagues first demonstrated a significant correlation between increased Treg numbers and MDS subgroups with ≥5% bone marrow blasts, high IPSS scores, and disease progression (Kordasti et al., 2007), consistent with our ecosystem state characterization identifying Treg-like transcriptional programs as components of the MDS immunosuppressive ecosystem.

The prognostic significance of Treg ecosystem expansion in MDS has been further refined by subsequent studies identifying specific Treg subsets with distinct functional properties. Mailloux et al. demonstrated that expansion of effector memory Tregs was significantly associated with anemia, reduced hemoglobin, and blast counts ≥5%, with independent prognostic importance for survival (Mailloux et al., 2012). Our ecosystem state transcriptomic data complement these findings by identifying gene expression signatures associated with Treg-like activation and suppressive function within the MDS immune ecosystem, including upregulation of immunosuppressive cytokines such as IL-10 and TGF-β1.

Our RT-PCR validation demonstrated increased IL-10 and TGF-β1 expression in the MDS-derived cell-line models, supporting the presence of immunoregulatory transcriptional programs. Chronic inflammatory signaling is recognized as an important component of MDS pathobiology, involving interconnected pathways including toll-like receptor signaling, inflammasome activation, and dysregulated NF-κB signaling (Sallman and List, 2019). Importantly, cytokine and chemokine expression detected by scRNA-seq and RT-PCR reflects transcript abundance; protein-level and secretion assays are required to establish functional cytokine production.

A key ecosystem finding is the detection of immune checkpoint gene expression patterns—including PDCD1, CTLA4, LAG3, and HAVCR2—that exhibited sparse but distinct expression across the MDS ecosystem state UMAP landscape. RT-PCR confirmed substantial upregulation of CD274 in MDS cell lines (5.7-fold in SKM-1, 3.9-fold in MUTZ-1), supporting activation of checkpoint-associated ecosystem programs. These observations are consistent with extensive literature documenting PD-1/PD-L1 axis dysregulation in MDS (Yang et al., 2014; Boddu et al., 2018; Yang et al., 2022). However, clinical trials incorporating immune checkpoint blockade in MDS have shown limited or non-superior efficacy compared with standard hypomethylating-agent-based treatment (Zeidan et al., 2022), suggesting that single-axis checkpoint blockade may be insufficient to overcome the complex immunosuppressive ecosystem networks operating in MDS. Our comprehensive characterization of checkpoint molecule expression across multiple immune ecosystem state cell types provides a foundation for developing more sophisticated combinatorial immunotherapeutic ecosystem-targeting strategies.

The CD274 perturbation experiments were designed to test cell-intrinsic regulatory effects in MDS-derived cell-line ecosystem models. The divergent IL-10 and FOXP3 responses following CD274 perturbation should be interpreted cautiously. The differences between SKM-1 and MUTZ-1, including their distinct baseline FOXP3 levels, indicate substantial cell-line-dependent heterogeneity. The present data do not establish a FOXP3-independent or IL-10-dominant mechanism; validation through co-culture systems, *in vivo* models, primary patient samples, and sorted immune-cell subsets is required to resolve this open mechanistic question regarding CD274-centered ecosystem regulation.

The role of Tregs in the MDS immune ecosystem appears to differ substantially between lower-risk and higher-risk disease, reflecting the biphasic nature of immune involvement in MDS pathogenesis. In lower-risk MDS, reduced Treg numbers in bone marrow are associated with expansion of cytotoxic T lymphocytes and immune-mediated suppression of hematopoiesis (Aggarwal et al., 2011). Conversely, in higher-risk MDS, Treg expansion creates an immunosuppressive ecosystem environment that facilitates malignant clone survival and disease progression (Kordasti et al., 2007; Mailloux et al., 2012). Kotsianidis et al. demonstrated that CD4^+^CD25+FOXP3+ Tregs in MDS patients show kinetic changes with disease evolution and maintained suppressive function despite quantitative alterations (Kotsianidis et al., 2009), consistent with the ecosystem-level Treg-like program redistribution observed in our T/NK ecosystem state analysis.

Emerging evidence suggests that immune ecosystem profiles possess independent prognostic value beyond traditional risk stratification systems. Kahn et al. demonstrated that an immunosuppressive ecosystem characterized by high percentages of Tregs and low percentages of progenitor B cells was associated with poorer survival in lower-risk MDS patients (Kahn et al., 2015). Leone et al. further showed that reduced Treg numbers in bone marrow preferentially associate with expansion of cytotoxic T lymphocytes in low-risk MDS patients (Leone et al., 2022). These findings underscore the importance of ecosystem state characterization—including T/NK-cell state remodeling and immunosuppressive communication networks—for prognostic and therapeutic stratification in MDS precision oncology.

Our mapping of cell-cell ecosystem communication patterns illustrates the transcriptome-based communication architecture linking T cells, B cells, NK cells, monocytes, DCs, and neutrophils within the MDS bone marrow ecosystem. The dual role of the immune ecosystem in MDS—initially attempting to suppress malignant clones but ultimately facilitating their expansion—represents a fundamental challenge for therapeutic intervention. Our ecosystem-level characterization of CD274-centered communication networks, immunosuppressive T/NK state remodeling, and multi-cellular checkpoint expression provides a framework for identifying ecosystem-informed combination strategies that target the immunosuppressive MDS ecosystem at multiple levels simultaneously.

### Limitations

Several important limitations of the current exploratory ecosystem-state study must be acknowledged. The relatively small total cell number and low cell counts in some clusters constrain statistical power, particularly for rare populations, differential expression, and ligand-receptor inference. First, the scRNA-seq analysis was based on public datasets with limited patient-level clinical annotation. Available metadata from GSE241417 and GSE120221 are summarized in Supplementary Table S1; however, MDS subtype, IPSS-R risk category, age, sex, treatment timing, treatment response, and longitudinal clinical follow-up were incomplete for the MDS samples. Therefore, the current ecosystem state analysis was conducted at the disease-context and sample-context levels rather than as a subtype-specific, treatment-stage-specific, or outcome-associated study.

Second, although the expanded analyses using GSE120221 as an external healthy bone marrow reference provided broader disease-comparison ecosystem context, cross-dataset effects should be considered when interpreting MDS-control ecosystem comparisons. These analyses were framed as descriptive and hypothesis-generating rather than definitive population-level conclusions, because residual cross-dataset technical effects may remain after harmonization.

## Conclusion

This study presents a computational single-cell transcriptomic characterization of the MDS bone marrow immune ecosystem, resolving eleven transcriptionally distinct Leiden-defined populations/states with differences in immune-cell composition, checkpoint expression, cytokine-related programs, and inferred intercellular communication. Cross-dataset MDS-versus-healthy comparisons are descriptive and hypothesis-generating because residual technical confounding cannot be excluded. CD274-high cells were associated with immunoregulatory module scores, and CD274-centered ligand-receptor analyses identified computationally inferred communication patterns rather than experimentally established signaling mechanisms. In MDS-derived cell-line models, CD274 perturbation was associated with heterogeneous changes in IL-10 and FOXP3, without establishing direct molecular regulation. These findings provide a hypothesis-generating framework for future validation in larger clinically annotated cohorts, primary patient-derived immune subsets, and mechanistic multicellular models.

## Data Availability

The datasets analyzed in this study are publicly available in the Gene Expression Omnibus under accession numbers GSE241417 and GSE120221.
